# Efficacy and safety of traditional Chinese medicines for non-alcoholic fatty liver disease: a systematic literature review of randomized controlled trials

**DOI:** 10.1186/s13020-020-00422-x

**Published:** 2021-01-11

**Authors:** Zuanji Liang, Xianwen Chen, Junnan Shi, Hao Hu, Yan Xue, Carolina Oi Lam Ung

**Affiliations:** State Key Laboratory of Quality Research in Chinese Medicine, Institute of Chinese Medical Sciences, University of Macau, Taipa, Macao

**Keywords:** Non-alcoholic fatty liver disease, Traditional Chinese medicine, Randomized clinical trial, RCT, CONSORT-CHM, Risk of bias

## Abstract

**Background:**

Non-alcoholic fatty liver disease (NAFLD) is a common liver disease that may progress into, in the absence of proper treatment, severe liver damage. While the optimal pharmacotherapy for NAFLD remains uncertain and the adherence to lifestyle interventions is challenging, the use of herbal medicines such as traditional Chinese medicines (TCMs) to manage the condition is common. The evidence about TCMs in the management of NAFLD is continuously developing through randomized controlled trials (RCTs). This study aims to identify and evaluate the emerging evidence about the efficacy and safety of TCMs for NAFLD.

**Methods:**

A systematic literature search was conducted to identify RCTs which investigated TCMs in the management of NAFLD published in 6 electronic databases including PubMed, the Cochrane Library, EMBASE, Web of Science, Scopus and China National Knowledge Infrastructure since inception to September 2020. RCTs comparing TCMs with no treatment, placebo, non-pharmacological and/or pharmacological interventions were included irrespective of language or blinding. The quality of reporting was evaluated using the Consolidated Standards of Reporting Trials Statement extensions for Chinese herbal medicine Formulas (CONSORT-CHM). Risk-of-bias for each study was assessed using the Cochrane risk of bias tool.

**Results:**

A total of 53 RCTs involving 5997 participants with NAFLD were included in this review. Each included RCT tested a different TCMs giving a total of 53 TCMs identified in this study. Based on the evaluation of the RCT results, TCMs might have various beneficial effects such as improving TCM syndrome score, liver function, and body lipid profile. A range of non-serious, reversible adverse effects associated with the use of TCMs were also reported. However, no conclusion about the efficacy and safety of TCMs in NAFLD can be made. The quality of reporting was generally poor and the risks of bias was mostly uncertain in all trials.

**Conclusions:**

There is some evidence from RCTs that supported the effectiveness and safety of TCMs for NAFLD. However, no conclusive recommendations can be made due to the questionable quality of the RCTs. Improvement in the RCT protocol, the use of a larger sample size, a setting of multicenter, and a more focused approach in selecting TCMs are recommended for developing high quality evidence about the use of TCMs in managing NAFLD.

## Background

Non-alcoholic fatty liver disease (NAFLD) is a common chronic liver disease that refers to liver steatosis in the absence of significant alcohol consumption, use of susceptible medication, or other pre-existing liver condition or infections that result in fat accumulation [1]. Metabolic risk factors are the majority causes of the NAFLD, such as obesity, diabetes mellitus, and dyslipidemia [1]. The global prevalence of NAFLD was estimated at 9.7% and an increasing trend had been shown in recent years [2]. NAFLD has a wide spectrum of liver diseases ranging from simple liver steatosis to more advanced forms, such as nonalcoholic steatohepatitis, progressive fibrosis and cirrhosis [3]. This progression may be slowed or even reversed with proper management. However, at present, the optimal pharmacotherapy for NAFLD remains uncertain as no pharmacological agent has been officially approved for treating NAFLD [4]. The best recommendations for NAFLD patients were lifestyle improvement and physical exercise [5, 6]. Lifestyle recommendation such as dietary restriction and enhanced physical activity have shown to beneficial in improving hepatic pathologic syndrome and reducing hepatic fat accumulation. However, adherence to lifestyle changes has been shown to be challenging. In the absence of proper management, more serious inflammation and degeneration of liver cells may result leading to irreversible liver injury and thus increasing the risks of hepatocellular carcinoma development [7].

In light of limited treatment options for NAFLD, herbal medicines, with traditional Chinese medicines (TCMs) in particular, have become an increasingly common healthcare choice for patients to manage or even treat the disease [8]. TCMs has a long history of use to treat liver disease in China. According to the Chinese traditional medical theory, NAFLD may result when Gan-collaterals are obstructed by the dysfunction of Gan-qi catharsis and Pi transportation, internal accumulation of dampness-heat and dirty phlegm, and blocking of blood stasis [9]. Therefore, TCMs are used mainly to channel Gan-qi, promote blood circulation, reduce phlegm and cleanse unclean elements. In modern science, preclinical and clinical studies suggested that TCMs may be an effective measure for NAFLD due to the beneficial effects on fatty acid metabolism that improves lipid metabolic parameters such as decreased levels of triglycerides, total cholesterol and low-density lipoprotein, alanine aminotransferase, aspartate aminotransferase and increased production of high-density lipoprotein [4]. Such TCMs often contain complex constituents with multiple pharmacological activities at various targets. For instance, Qushi Huayu decoction, a well-known TCM formula consisting of at least 5 medicinal plants, has been reported to effectively reverse elevated levels of free fatty acid and total triglycerides, and improve hepatic steatosis and inflammation through multiple signaling pathways [10, 11]. At the same time, TCMs, like all pharmacological agents, may potentially have the risks of adverse effects and toxicity which should also be determined and taken into consideration when deciding on the treatment option for NAFLD [12].

In recent years, increasing number of RCTs have been conducted to investigate the benefits and risks of TCMs in treating NAFLD. A systematic review conducted in 2013 assessed the benefits and risks of herbal medicines (including TCMs) for people with NAFLD and reported that some of the herbal preparations investigated seemed to have positive efforts on selected clinical indicators without inducing increased risks of adverse effects compared to the control groups [12]. Subsequent studies further suggested various underlying mechanisms through which herbal medicines might prevent NAFLD [4, 13]. The evidence from RCTs or other studies about TCMs for NAFLD is mounting but, nevertheless, inconclusive if not conflicting. To keep track of the emerging evidence about TCMs in NAFLD, there needs to be continuous efforts to critically appraise the evidence about the efficacy and adverse effects of TCMs. Therefore, the study aimed to conduct a systematic review of RCTs which investigated the use of TCMs in NAFLD in order to evaluate the benefits and harms of TCMs for patients with NAFLD. In addition, by assessment the quality of RCTs on TCMs for NAFLD, further analysis would be carried out to explore how to improve the RCT design and the reporting of RCT findings in relation to TCMs. The findings will be useful for updating the current knowledge about TCMs in NAFLD, informing patients’ choice of management measures, and identifying areas in need of further research.

## Methods

This systematic review was conducted in compliance with the Preferred Reporting Items for Systematic Reviews and Meta analyses (PRISMA-P guidelines) [14]. To evaluate the quality and risk bias of the studies, the Cochrane Collaboration’s Risk of Bias tool [15] and the Consolidated Standards of Reporting Trials Statement extensions for Chinese herbal medicine Formulas (CONSORT-CHM) [16] were used in this review. CONSORT-CHM had been developed as one of the extensions of the Consolidated Standards of Reporting Trials (CONSORT) Statement to set the baseline for reporting trials using CHM formulas [17]. In addition to the basic criteria of reporting clinical trials as listed in the CONSORT, the CONSORT-CHM had additional consideration that adequately took into account the unique characteristics of TCM—theory, principles, formulas, and Chinese medicinal substances.

### Types of studies

Randomized, double or triple-blinded, controlled trials which investigated the use of TCMs in NAFLD irrespective of blinding, publication status or date of publication, published in English or Chinese were considered for inclusion in this study. Quasi-randomized and observational studies were excluded. In this review, TCMs encompassed preparations which might include the use of the plant, animal materials, and mineral substances in preparations administered as capsules, tablets, teas, decoctions, granules and powders according to the unique principles and comprehensive theory of Traditional Chinese Medicine. In additional, TCM preparations which contained TCMs listed in TCM related standards such as the Chinese Pharmacopoeia [18] or the Grand Dictionary of Chinese Medicine [19] were eligible for consideration in this review.

### Types of RCT participants

Participants of any age, gender or ethnic origin with a clear diagnosis of NAFLD irrespective of the diagnostic method, diabetic status or the presence of non-alcoholic steatohepatitis (NASH) were eligible for the studies. We excluded RCTs in which participants recorded had viral hepatitis, liver function decompensation, other liver diseases or undergone liver transplantation previously.

### Types of interventions

For inclusions, RCTs which compared TCMs alone or TCMs in combination with behavioral interventions against placebo, no treatment, pharmacological therapy and/or other behavioral interventions were considered. Behavioral interventions referred to lifestyle interventions such as dietary modification and/or exercise regime. Pharmacological therapies referred to any other medicinal herbs not considered TCMs or conventional medicines such as prescription medicines regardless of the mechanisms of actions.

### Types of outcome

In order to address the objectives of this study, both efficacy and safety of the TCMs investigated in the RCTs included in this review were to be analyzed. As such, the primary and the secondary outcomes of managing NAFLD with the use of TCMs included the following:

#### Primary outcomes

The primary outcome measures considered included changes in TCM syndrome score and experiences in adverse reactions. According to the Chinese Medicine Clinical Research of New Drugs Guiding Principles [20], TCM syndrome score is the scoring method to evaluate patients symptoms such as dry mouth, bitter eyes, dry eyes, bleeding gums, insomnia and dreams, abdominal distension, loss of appetite, fatigue, loss of appetite, hypochondriac pain, waist and knee pain, urine and bowel, etc. The symptoms could be rated as “no”, “light”, “moderate” or “severe” represented by the score of 0 point, 1 point, 2 points, and 3 points respectively.

On the other hand, adverse reactions experienced during or immediately after the intervention duration would be considered. Depending on the availability of data, adverse events would be classified as serious or non-serious. A serious adverse reaction was defined as any effects that could increase mortality; was life-threatening; required hospitalization; resulted in persistent or significant disability; caused a congenital anomaly or birth defect, or any important medical event that might have jeopardized the health of the patients. Non-serious adverse events, on the other hand, referred to any untoward medical occurrence not necessarily having a causal relationship with the treatment, but resulting in a dose reduction or discontinuation of treatment (at any time after commencement of treatment) [21].

#### Secondary outcomes

The secondary outcome measures were considered as the followings [12]:

1. Radiological response which indicated the degree of fatty liver infiltration assessed by B-ultrasound, computed tomography, magnetic resonance spectroscopy (MRS), or other imaging techniques).
2. Liver function as reflected by the serum activities of aspartate aminotransferase (AST), alanine aminotransferase (ALT), alkaline phosphatases (ALP), gamma-glutamyl-transpeptidase (GGT), total bilirubin (TB), direct bilirubin (DB), total serum protein (TP), albumin (ALB), or albumin/globulin ratio (A/G).
3. Indicators of body weight which might include the body mass(BM), body mass index (BMI), and waist hip ratio (WHR).
4. Blood sugar which referred to the fasting blood glucose (FBG), fasting plasma insulins (FINs), and homeostasis model assessment-estimated insulin resistance (HOMA-IR).
5. Blood lipids such as serum activities of triglyceride (TG), total cholesterol (TC), high density lipoprotein-cholesterol (HDL-C), low density lipoprotein-cholesterol (LDL-C), and cholesterol (CHOL).
6. Other secondary outcome might include diamine oxidase (DAO), lipopolysaccharide, D-lactic acid, Claudin-1, Claudin-4, high sensitivity C-reactive protein (hs-CRP), tumor necrosis factor-α and so on.

### Search methods of studies

#### Electronic searches

This systematic review was performed according to the PRISMA-P guidelines [22] for searching the literature. Six electronic databases including PubMed, the Cochrane Library, EMBASE, Web of Science, Scopus and China National Knowledge Infrastructure (CNKI) were searched for RCTs evaluating TCMs in the management or treatment of NAFLD from inception to September 2020. The three primary search terms were “NAFLD”, “TCM” and “RCT”. As shown in Table 1, the operational definition used for these three primary terms referred to the related vocabularies. MeSH terms and keywords were used to develop a comprehensive search strategy and to ensure the validity of the strategy. Terms within “NAFLD”, “TCM”, and “RCT” were combined with OR, and the following results from each concept were combined with AND. The references of the included studies and Cochrane reviews on NAFLD were also searched.

**Table 1:** Search term identifiers

Category	Entry search terms in English	Entry search terms in Chinese
RCT	Clinical	隨機AND對照AND臨床試驗
	Trial^a^	隨機AND對照AND臨床研究
		隨機AND對照AND臨床觀察
NAFLD	"Nonalcoholic fatty"	非酒精性脂肪肝
	"Non-alcoholic fatty"	非酒精性脂肪性肝病
	"Non-alcoholic fatty liver disease" [mesh]	非酒精性脂肪性肝炎
	Nonalcoholic AND "fatty liver" [mesh]	
	Non-alcoholic AND "fatty liver" [mesh]	
	NAFLD	
	"Nonalcoholic steatohepatitis"	
TCM	Phytotherapy	中醫
	Herbal medicine^a^	中藥
	Plant preparation^a^	草藥
	Chinese medicine^a^	
	Complementary medicine^a^	
	“Drugs, Chinese herbal” [Mesh]	
	“Medicine, Chinese traditional” [Mesh]	
	“Medicine, traditional” [Mesh]	
	“Plant preparations” [Mesh]	
	Medicinal plant^a^	
	Plant medicinal product^a^	
	Herb^a^	

AND retrieves results that include all the search terms

^a^Including but not limited to

#### Exclusion criteria and screening

The title, abstract and full text of each study were screened for meeting the inclusion criteria. The process of screening for inclusion consisted of 2 rounds of assessment. In Round 1, the title and the abstract were studied to exclude non-TCMs related RCTs. The following studies were also excluded from the first round of screening (1) review, meta-analysis, protocol; (2) non-randomized trial; (3) pharmacodynamics or pharmacology studies; (4) acupuncture studies; (5) other disease studies or NAFLD combined other diseases studies; (6) studies about vitamin or mineral or fortified food or probiotic; (7) studies about ingredients extracted from plants or herbs not listed in TCM related standards; (8) studies about the ingredient or component listed in the TCM related standards [18, 19] but was used in combination with other dietary supplements or nutraceuticals. In Round 2, full-text review was conducted to exclude non-TCM-related RCTs and those RCTs which tested the effects of TCMs in combination with pharmacological interventions in the test group.

### Data collection and analysis

#### Selection of studies

The title and abstract was separately screened by 2 of the authors (ZL, XC) according to the inclusion criteria outlined above. Full texts of potentially relevant articles were retrieved for detailed assessment. The Cochrane evaluations and CONSORT-CHM statement evaluation were independently performed by two of the authors (ZL, XC) following the guidelines, and the disagreements were discussed and resolved by discussion or consultation with two other authors (JS, COLU).

#### Data extraction and management

Endnote X9 was used to categorize and file all the references. Excel 2013 was used to extract data and record. A standard extraction form was used to extract relevant data from the eligible trials, which contained the basic information of study, methods, intervention, participants, outcomes, overall findings, etc. The main information extracted from each included studies for further analysis is listed in the following:

- Basic information of study
  - First author and publication year; and
  - Language.

- Methods
  - Trial design;
  - Date of trial;
  - Setting of trial;
  - Inclusion and exclusion of patients; and
  - Diagnosed criteria (integration medicine or western medicine).

- Intervention
  - Treatment drugs;
  - Using does;
  - Interment duration;
  - lifestyle intervention; and
  - Follow-up period.

- Participants
  - The number of participants randomized;
  - The number of participants analyzed;
  - Sex ratio, history of NFALD, mean age; and
  - Dropouts.

- Outcomes
  - Primary outcomes;
  - Secondary outcomes;

- Overall findings
  - Efficacy rate;
  - Statistical difference in the efficacy;
  - Safety information.

#### Assessment of risk of bias in included studies

Two of the authors (ZL, XC) evaluated the risk of bias of each trial independently in accordance with the Cochrane Handbook for Systematic Reviews of Interventions [15] and the CONSORT-CHM [23]. All criteria were referred to from the Cochrane guidelines. The Cochrane risk-of-bias tool was used to assess all trials’ quality. There were three categories [17] of the results: “low risk of bias”, “Unclear risk of bias” and “High risk of bias”. The judgement was made based on the definitions on recommendations from these two assessment methods as shown in the following.

- Sequence generation
  - Low risk of bias: clearly indicate the method used (e.g., table of random numbers, random block or computer random number generation);
  - Unclear risk of bias: no specified information about the methods; and
  - High risk of bias: no mentioned about the methods.

- Allocation concealment
  - Low risk of bias: the participants were allocated table of random numbers, random block or computer random number generation;
  - Unclear risk of bias: no specified information about the allocation methods (e.g. only reported the ratio of different groups); and
  - High risk of bias: no mentioned about the allocation methods.

- Blinding
  - Low risk of bias: the study authors described the trial as blinding and mentioned the methods of blinding;
  - Unclear risk of bias: the study authors mentioned used blinding methods without any descriptions of blinding; and
  - High risk of bias: no mentioned about the blinding and the methods.

- Incomplete outcome data
  - Low risk of bias: the study authors reported the number of dropouts and withdrawals, and described the reason or reported there was no dropouts or withdrawals;
  - Unclear risk of bias: the study author reported there was dropout or withdrawals without any specified information; and
  - High risk of bias: no mentioned about dropout or withdrawals.

- Selective outcome reporting
  - Low risk of bias: the study authors reported predefined, or clinically relevant and reasonably expected outcomes;
  - Unclear risk of bias: the study authors did not report all predefined, or clinically relevant and reasonably expected outcomes, or the data reported did not match the methods; and
  - High risk of bias: no mentioned about the predefined, or clinically relevant and reasonably expected outcomes.

- Other bias
  - Low risk of bias: the trial appeared no other components that may cause the risk of bias;
  - Unclear risk of bias: the trial may or may not have other components that may cause the risk of bias. and
  - High risk of bias: there were other factors in the trial that could put it at risk of bias (e.g. the differences of baseline, for-profit involvement, and inappropriate intervention design).

#### Quality assessment methods

Each article included was independently assessed by two of the authors (ZL, XW). Disagreements were settled through discussion or consultation with the other two authors (JS, COLU). The 25-item version of the CONSORT CHM statement was used to assess for the quality of the trials included. The checklist provides a set of guidelines that may be used to identify the strengths and weaknesses of clinical trials for the treatments of TCM intervention. To measure compliance, a grading system was devised for each criterion, where the reviewer gave a score of “0” if the item was not present at all, a “1” if the feature was partially present, for instance, some aspects of the CONSORT item were missing or being described unclearly, and a “2” if the item was present and clear. By applying the CONSORT criteria for all relevant sections of each study, an overall summary of the reporting quality of the included RCTs was produced. The evaluation method and results were independently checked for validity and consistency by all 6 authors.

## Results

### Search results

**Fig. 1:**
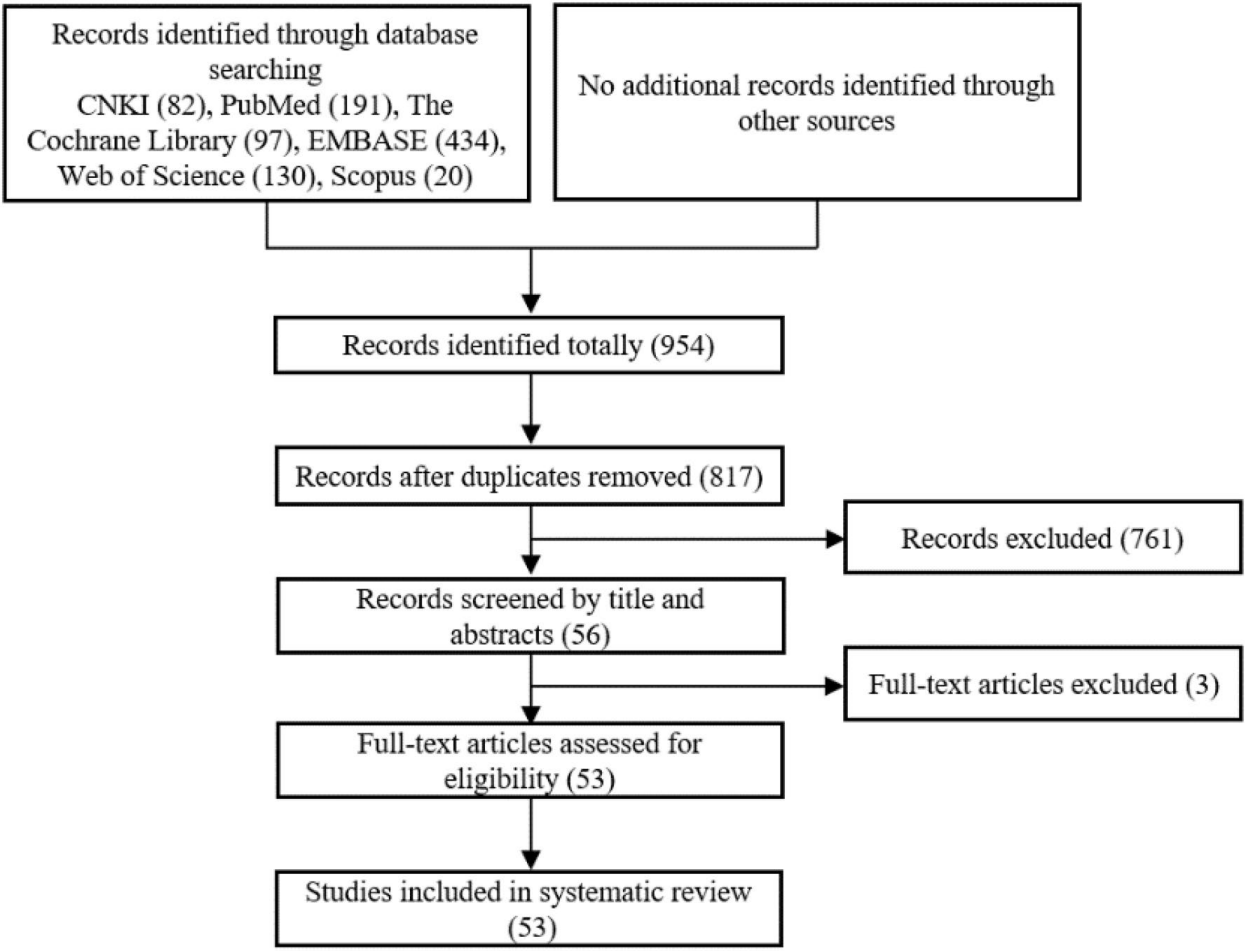
PRISMA flow-chart of study selection

The screening process conducted in accordance with the PRISMA guidelines is summarized in the flow diagram as shown in Fig. 1. A total of 954 citations were identified in the initial searches from the selected electronic databases and related sources. After the removal of duplications, 817 potentially relevant articles were retained for further assessment. Due to a range of reasons, 761 records were further excluded after Round 1 of the screening process (reading titles and abstracts): not randomized trials, review or meta-analysis or protocol articles, pharmacodynamics or pharmacology studies, acupuncture studies, other disease studies, or combined other diseases studies, or herbal ingredients not listed in the TCMs-related standards [18, 19], or the use of TCMs was combined with other pharmacological agents. Fifty-six articles were then retrieved and included for further assessment. After accessing and reviewing the full text, 3 more articles were excluded including 2 studies that investigated non-TCMs interventions and 1 study which used both TCMs and chemical drugs in the test group. Eventually, 53 eligible articles published in Chinese (n = 48) and English (n = 5) were included in this review.

### Description of studies

Among the 53 publications included in this review, 48 were published in Chinese and only 5 were published in English [24–28] among the included trials, one trial was conducted in Korea [28] and the remaining 52 trials were conducted in China. All of the included studies set a parallel two-arm design. More details are showed in Table 2.

**Table 2:** Basic descriptions of the studies included in this review

No	Authors, year	TCM name in the test group	TCM component	Diagnosis criteria of NAFLD		History of NAFLD	Number of participants in total (test group, control group)	Number of male, female	Participants’ mean age (years old)
1	Wu et al., 2020 [43]	Jianpi Qingzhi prescription	Atractylodis Macrocephalae Rhizoma, Alismatis Rhizoma, Crataegi Fructus, Curcumae Radix, Poria, Cassiae Semen, etc.	IM		T: 3.05 ± 0.61 years C: 2.96 ± 0.67 years	60 (30, 30)	35, 25	T: 49.22 ± 5.65 C: 48.96 ± 5.71
2	Lyv et al., 2020 [47]	Shenge formula	Codonopsis Radix, Puerariae Lobatae Radix, Atractylodis Rhizoma, Atractylodis Macrocephalae Rhizoma, Salviae Miltiorrhizae Radix Et Rhizoma, etc.	WM		N/A	65 (34, 31)	37, 28	T: 42.03 ± 13.91 C: 44.94 ± 13.09
3	Yang et al., 2019 [59]	Yanggan Jieyu granule	Moutan Cortex, Gardeniae Fructus, Angelicae Sinensis Radix, Paeoniae Radix Alba, Radix Bupleuri, Poria, etc.	IM		N/A	65 (30, 30)	37, 28	T: 42.03 ± 13.91 C: 44.94 ± 13.09
4	Yang et al., 2019 [97]	Qinjiang Baoling decoction	Radix Bupleur, Paeoniae Radix Alba, Gynostemmatis Herba, Schisandrae Chinensis Fructus, Curcumae Rhizoma, Poria, etc.	IM		T: 21.3 ± 13.4 months C: 20.9 ± 12.7 months	100 (50, 50)	59, 41	T: 57.3 ± 12.2 C: 51.7 ± 10.3
5	Li et al., 2018 [62]	Qutan Huoxue decoction	Atractylodis Macrocephalae Rhizoma, Gynostemmatis Herba, Curcumae Longae Rhizoma, Rhodiolae Crenulatae Radix Et Rhizoma, etc.	IM		N/A	140 (63, 62)	113, 27	T: 42.01 ± 9.60 C: 41.43 ± 9.31
6	Chen et al., 2017 [98]	Shuangqing granule	Gynostemmae Herba, Salviae Miltiorrhizae Radix Et Rhizoma, Polygonum Cuspidati Rhizoma Et Radix, Artemisia Scopariae Herba, and Nelumbinis Folium	IM		T: 0.5–8 years C: 0.6–7 years	103 (78, 25)	49, 53	Range: 18–65
7	Hu et al., 2017 [31]	Jianpishugan and Huoxuehuatan formulae	Astragali Radix, Radix Bupleuri, Atractylodis Macrocephalae Rhizoma, Alismatis Rhizoma, Cassiae Semen, Angelicae Sinensis Radix, etc.	IM		T: 1–12 years C: 1–13 year	140 (70, 70)	83, 57	T: 42.9 ± 5.3 C: 43.2 ± 5.7
8	Jiang et al., 2017 [41]	Dahuang Lidan capsule	Rhei Radix Et Rhizoma, Phyllanthi Fructus and Radix Gymnadeniae	IM		N/A	144 (72, 72)	104, 40	T: 43.0 ± 0.03 C: 43.4 ± 7.60
9	Yang et al., 2017 [53]	Cigu Xiaozhi capsule	Cremastrae Pseudobulbus, Alismatis Rhizoma, Crataegi Fructus, Salviae Miltiorrhizae Radix Et Rhizoma, Radix Bupleuri, Pinelliae Rhizoma Praeparatum, Cassiae Semen, etc.	WM		N/A	87 (43, 44)	50, 37	T: 49.02 ± 11.19 C: 50.63 ± 10.97
10	Jeong et al., 2017 [28]	HL tablet	Extract of *Magnolia officinalis*	WM		N/A	72 (22, 23, 23)*	54, 18	H: 39.1 ± 9.5 L: 45.5 ± 11.5 C: 42.7 ± 11.2
11	Sun et al., 2017 [68]	Jianpi Qushi prescription	Codonopsis Radix, Poria, Microctis Folium, Microctis Folium, Crataegi Fructus, Cassiae Semen, Pogostemonis Herba, etc.	IM		T: 3.41 ± 2.56 years C: 4.02 ± 2.73 years	120 (60, 60)	67, 53	T: 34.61 ± 12.47 C: 36.37 ± 10.25
12	Niu, 2016 [48]	Shugan Jianpi Huatan decoction	Codonopsis Radix, Atractylodis Macrocephalae Rhizoma, Poria, Alismatis Rhizoma, Coicis Semen, Artemisia Scopariae Herba, Radix Bupleuri, Curcumae Radix, Salviae Miltiorrhizae Radix Et Rhizoma, etc.	IM		T: 3.3 ± 0.4 years C: 3.5 ± 0.8 years	120 (60, 60)	72, 48	T: 47.2 ± 2.7 C: 46.4 ± 2.4
13	Zhang et al., 2016 [99]	Yiqi Qizhu decoction	Atractylodis Macrocephalae Rhizoma, Astragali Radix, Poria, Citri Pericarpium Reticulatae, Radix Bupleuri, Curcumae Radix, Coicis Semen, Pogostemonis Herba, etc.	IM		N/A	104 (52, 52)	67, 37	T: 24–60 C: 25–58
14	Zhang et al., 2016 [34]	Jiang Zhi granules	Gynostemmatis Herba, Polygonum Cuspidati Rhizoma Et Radix, Artemisia Scopariae Herba, Salviae Miltiorrhizae Radix Et Rhizoma and Nelumbinis Folium	IM		N/A	144 (73, 71)	110, 34	T: 42.4 ± 11.6 C: 44.8 ± 11.4
15	Qi et al., 2016 [63]	Jiangpi Shugan Huoxue Huatan decoction	Radix Bupleuri, Curcumae Radix, Atractylodis Macrocephalae Rhizoma, Pinelliae Rhizoma Praeparatum, Artemisia Scopariae Herba, Alismatis Rhizoma, Cassiae Semen, Salviae Miltiorrhizae Radix Et Rhizoma, Angelicae Sinensis Radix, Crataegi Fructus, etc.	IM		N/A	90 (45, 45)	53, 37	T: 49.49 ± 9.91 C: 48.87 ± 9.06
16	Tan et al., 2016 [100]	Shugan Jianpi decoction	Radix Bupleuri, Aurantii Fructus, Atractylodis Macrocephalae Rhizoma, Poria, Crataegi Fructus, Cyperi Rhizoma, Alismatis Rhizoma, Salviae Miltiorrhizae Radix Et Rhizoma, Citri Pericarpium Reticulatae, etc.	WM		T: 5.75 ± 2.41 years C: 5.43 ± 2.85 years	92 (45, 47)	50, 42	T: 40.25 ± 4.78 C: 43.02 ± 5.48
17	Yu et al., 2016 [69]	Qinggan Jiangzhi decoction	Poria, Atractylodis Macrocephalae Rhizoma, Salviae Miltiorrhizae Radix Et Rhizoma, Curcumae Longae Rhizoma, Cassiae Semen, Alismatis Rhizoma, etc.	IM		T: mean = 7.5 years C: mean = 8.2 years	125 (62, 63)	71, 54	T: 48.28 C: 49.81
18	Yin et al., 2016 [35]	Danxia Zhifang decoction; Simulated Zhiganxiao capsule	Salviae Miltiorrhizae Radix Et Rhizoma, Pinelliae Rhizoma Praeparatum, Radix Bupleuri, Poria, Citri Pericarpium Reticulatae, Raphani Semen, Crataegi Fructus, etc.	IM		N/A	202 (98, 104)	149, 53	T: 43.38 ± 9.75 C: 44.49 ± 9.80
19	Zhu, 2015 [33]	Qingdan Jiangzhi decoction	Artemisia Scopariae Herba, Poria, Atractylodis Macrocephalae Rhizoma, Citri Pericarpium Reticulatae, Nelumbinis Folium, Crataegi Fructus, Curcumae Radix, Alismatis Rhizoma, Radix Bupleuri, etc.	IM		T: 5.1 ± 1.2 years C:4.9 ± 1.3 years	80 (40, 40)	43, 37	T: 44.6 ± 9.6 C: 42.8 ± 8.4
20	Li, 2015 [64]	Huatan Lishi Huoxue granule combined granule decoction	Alismatis Rhizoma, Salviae Miltiorrhizae Radix Et Rhizoma, Crataegi Fructus, Cassiae Semen and Radix Bupleuri	N/A		T: 3.5 ± 2.2 years C: 3.9 ± 2.3 years	104 (52, 52)	78, 26	T: 42.3 ± 8.4 C: 41.7 ± 7.8
21	Chen et al., 2015 [101]	Zaozhu Yinchen decoction	Gleditsiae Spina, Atractylodis Macrocephalae Rhizoma, Artemisia Scopariae Herba, Rhei Radix Et Rhizoma and Gardeniae Fructus	IM		T: 30.32 ± 6.98 months C: 31.14 ± 7.21 months	78 (40, 38)	41, 37	T: 38.28 ± 4.82 C: 39.53 ± 4.62
22	Yu et al., 2015 [52]	Tiaogan Lipi recipe	Astragali Radix, Atractylodis Macrocephalae Rhizoma, Coicis Semen, Salviae Miltiorrhizae Radix Et Rhizoma, Cyperi Rhizoma, Radix Bupleuri, Artemisia Scopariae Herba, Polygonum Cuspidati Rhizoma Et Radix, Cassiae Semen, Crataegi Fructus, etc.	IM		T: 3.38 ± 4.17 years C: 3.61 ± 4.93 years	99 (66, 33)	52, 47	T: 49.22 ± 11.08 C: 49.34 ± 10.77
23	Li et al., 2015 [58]	Qinghua decoction	Nelumbinis Folium, Gynostemmatis Herba, Coicis Semen, Poria, Citri Pericarpium Reticulatae, Alismatis Rhizoma, Salviae Miltiorrhizae Radix Et Rhizoma, Atractylodis Macrocephalae Rhizoma, etc.	WM		T: 3.15 ± 1.52 years C: 3.82 ± 1.3 years	119 (60, 59)	74, 45	T: 51.1 ± 9.8 C: 48.8 ± 11.2
24	Liu et al., 2014 [49]	Heze lipid-lowering oral liquid	Gynostemmatis Herba, Atractylodis Macrocephalae Rhizoma, Salviae Miltiorrhizae Radix Et Rhizoma, Alismatis Rhizoma, Nelumbinis Folium, Citri Pericarpium Reticulatae, Crataegi Fructus, etc.	IM		Range: 0.5–10 years	120 (60, 60)	55, 65	T: 50 ± 12.27 C: 50.88 ± 13.17
25	Liu et al., 2014 [50]	Ruangan Jiangzhi capsule	Radix Bupleuri, Rhei Radix Et Rhizoma, Prunus Semen, Paeoniae Radix Alba, Nelumbinis Folium, Salviae Miltiorrhizae Radix Et Rhizoma, Crataegi Fructus, Atractylodis Macrocephalae Rhizoma, Astragali Radix, Schisandrae Chinensis Fructus, etc.	IM		T: mean = 2.9 years C: mean = 2.8 years	120 (60, 60)	87, 33	T: 45.7 C: 44.3
26	Li et al., 2014 [51]	Jianpi Shugan recipe	Gynostemmae Herba, Curcumae Radix, Atractylodis Macrocephalae Rhizoma, Poria, Alismatis Rhizoma, Salviae Miltiorrhizae Radix Et Rhizoma, Crataegi Fructus, etc.	IM		N/A	201 (100, 101)	143, 58	T: 43.41 ± 10.80 C: 39.81 ± 11.79
27	Zhu et al., 2014 [102]	Zhigan pill	Alismatis Rhizoma, Salviae Miltiorrhizae Radix Et Rhizoma, Cassiae Semen, Crataegi Fructus, Curcumae Radix, Polygonum Cuspidati Rhizoma Et Radix, etc.	IM		T: 3.72 ± 2.53 years C: 3.00 ± 2.35 years	158 (120, 38)	122, 36	T: 46.15 ± 7.37 C: 45.70 ± 9.41
28	Wen et al., 2014 [60]	Jiangan Xiaozhi granule	Curcumae Rhizoma, Curcumae Longae Rhizoma, Notoginseng Radix Et Rhizoma, Salviae Miltiorrhizae Radix Et Rhizoma, Astragali Radix, etc.	IM		T: 29.97 ± 10.96 months C: 30.14 ± 10.60 months	73 (37, 36)	35, 38	T: 43.10 ± 9.14 C: 43.08 ± 8.65
29	Deng, 2013 [103]	Jiangan Sanqing decoction	Lycii Fructus, Astragali Radix, Atractylodis Macrocephalae Rhizoma, Crataegi Fructus, Nelumbinis Folium, Citri Pericarpium Reticulatae, Pinelliae Rhizoma Praeparatum, Radix Bupleuri, Salviae Miltiorrhizae Radix Et Rhizoma, etc.	WM		T: 3.3 ± 1.2 years C: 3.1 ± 1.3 years	60 (30, 30)	40, 20	T: 44.1 ± 6.2 C: 43.3 ± 5.9
30	Li et al., 2013 [54]	Jianpi Huatan Zhuyu prescription	Artemisia Scopariae Herba, Crataegi Fructus, Curcumae Longae Rhizoma, Prunus Semen, Atractylodis Macrocephalae Rhizoma, Cassiae Semen, Raphani Semen, etc.	IM		T: 23.3 ± 11.1 months C: 21.4 ± 13.2 months	118 (60, 58)	59, 59	T: 43.2 ± 14.2 C: 42.7 ± 13.0
31	Song et al., 2013 [104]	Modified Linggui Zhugan decoction	Poria, Atractylodis Macrocephalae Rhizoma, Cinnamomi Ramulus, Glycyrrhizae Radix Et Rhizoma, Caulis Nelumbonis and Alismatis Rhizoma	IM		T: 4.24 ± 2.10 years C: 4.20 ± 2.12 years	90 (48, 42)	72, 18	T: 44.8 ± 4.2 C: 42.2 ± 4.9
32	Li et al., 2013 [42]	Qushi Huayu decoction	Artemisia Scopariae Herba, Polygonum Cuspidati Rhizoma Et Radix, Herba Hyperici Japonici, Curcumae Longae Rhizoma, and Gardeniae Fructus	IM		N/A	164 (72, 73)	82, 82	T: 44.6 ± 10.9 C: 45.6 ± 11.2
33	Wong et al., 2013 [26]	*Phyllanthus urinaria*	*Phyllanthus urinaria*	N/A		N/A	60 (40, 20)	33, 27	T: 52 ± 10 C: 48 ± 11
34	Pan et al., 2013 [27]	Jiang Zhi granule	Gynostemmatis Herba, Salviae Miltiorrhizae Radix Et Rhizoma, Polygonum Cuspidati Rhizoma Et Radix, Artemisia Scopariae Herba, Nelumbinis Folium	WM		T: 26.61 ± 29.29 m C: 30.02 ± 34.59 m	221 (111, 110)	177, 44	T: 42.39 ± 11.55 C: 44.82 ± 11.41
35	Chen et al., 2012 [45]	Jindanwang mixture	lysimachiae Herba, Sargassum, Plantaginis Semen, Salviae Miltiorrhizae Radix Et Rhizoma, Curcumae Radix, Artemisia Scopariae Herba, etc.	IM		Mean = 1.25 years	124 (62, 62)	79, 45	Range: 28–65
36	Zhao et al., 2012 [30]	Huatan Xiezhuo recipe	Alismatis Rhizoma, Cassiae Semen, Salviae Miltiorrhizae Radix Et Rhizoma, Crataegi Fructus, Radix Bupleuri, etc.	IM		N/A	194 (96, 98)	N/A	N/A
37	Wang et al., 2012 [65]	Yigan Jiedu decoction	Sargassum, Curcumae Radix, Cassiae Semen, Curcumae Longae Rhizoma, Sedi Herba, Ginseng Folium, Radix Bupleuri, etc.		IM	T: 4.8 ± 3.6 years C: 4.1 ± 4.3 years	90 (45, 45)	49, 41	T: 49.64 ± 14.29 C: 47.82 ± 15.08
38	Pei et al., 2012 [70]	Qinggan Huatan Huoxue recipe	Cassiae Semen, Radix Bupleuri, Citri Pericarpium Reticulatae, Pinelliae Rhizoma, Curcumae Radix, Crataegi Fructus, Rhei Radix Et Rhizoma, Artemisia Scopariae Herba, etc.		IM	T: 17.83 ± 19.55 months C: 20.05 ± 28.11 months	150 (78, 72)	101, 49	T: 43.44 ± 12.76 C: 44.56 ± 12.30
39	Wu, 2012 [83]	Jianpi Huatan Fang	Atractylodis Rhizoma, Atractylodis Macrocephalae Rhizoma, Poria, Alismatis Rhizoma, Curcumae Radix, Crataegi Fructus, Salviae Miltiorrhizae Radix Et Rhizoma, etc.		WM	T: 1.8 ± 1.2 years C: 2.1 ± 1.4 years	60 (30, 30)	53, 7	T: 9.3 ± 2.1 C: 8.9 ± 2.5
40	Tong et al., 2011 [105]	Jiedu Jianpi Tongluo decoction	Semen Hoveniae, Curcumae Longae Rhizoma, and Salviae Miltiorrhizae Radix Et Rhizoma		WM	Range: 1–8 years	100 (45, 46)	58, 42	36.9
41	Zhang et al., 2011 [66]	Kangzhi decoction	Astragali Radix, Crataegi Fructus, Chrysanthemi Flos, Atractylodis Macrocephalae Rhizoma, Cassiae Semen, Alismatis Rhizoma, Nelumbinis Folium, Atractylodis Rhizoma, Sedi Herba, etc.		IM	T: 0.3–12.5 years C: 0.3–11.5 years	182 (92, 90)	79, 103	T: 38.6 ± 12.65 C: 39.3 ± 13.47
42	Gu et al., 2011 [61]	Zhigan capsule	Alismatis Rhizoma, Poria, Artemisia Scopariae Herba, Nelumbinis Folium, Crataegi Fructus, Radix Bupleuri, Puerariae Lobatae Radix, Salviae Miltiorrhizae Radix Et Rhizoma, etc.		IM	T: 1–10 years C: 1–11 years	100 (50, 50)	70, 30	T: 41.2 ± 10.7 C: 41.6 ± 11.1
43	Zhao et al., 2010 [46]	Qiyin tea	Astragali Radix, Artemisia Scopariae Herba, Crataegi Fructus, Psoraleae Fructus, Alismatis Rhizoma, Cassiae Semen, Notoginseng Radix Et Rhizoma, Rhei Radix Et Rhizoma, etc.		IM	N/A	112 (56, 56)	46, 66	N/A
44	Ding et al., 2010 [88]	Pingganjian decoction	Astragali Radix, Typhae Pollen, Curcumae Longae Rhizoma,Coptidis Rhizomacoptidis Rhizoma, Alismatis Rhizoma, Poria, Artemisia Scopariae Herba, Crataegi Fructus, etc.		IM	N/A	105 (54, 51)	60, 45	T: 45.8 ± 13.5 C: 47.2 ± 15.6
45	Pan et al., 2010 [32]	Tiaozhiji granules	Curcumae Rhizoma, Curcumae Radix, Raphani Semen, Crataegi Fructus, Aurantii Fructus, Alismatis Rhizoma, etc.		IM	T: 24.1 ± 10.4 years C: 22.3 ± 12.1 years	70 (35, 35)	49, 21	T: 46.0 ± 13.7 C: 50.9 ± 16.4
46	Liu et al., 2008 [67]	Qiyin granules	Astragali Radix, Artemisia Scopariae Herba, Crataegi Fructus, Psoraleae Fructus, Alismatis Rhizoma, Cassiae Semen, etc.		IM	N/A	70 (35, 35)	N/A	N/A
47	Ji et al., 2008 [25]	Danning tablet	Radix Et Rhizoma Rhei, Giant Knotweed Rhizome, Pericarpium Citri Reticulatae Viride, Aurantii Nobilis Pericarpium, Radix Curcu Mae, Fructus Crataegi and Imperatae Rhizoma		IM	N/A	135 (102, 33)	102, 33	T: 48.37 ± 9.60 C: 44.43 ± 10.40
48	Chen et al., 2008 [29]	Xiaoyao San decoction and Danggui Shaoyao San decoction	Radix Bupleuri, Angelicae Sinensis Radix, Paeoniae Radix Alba, Atractylodis Macrocephalae Rhizoma, Poria, Alismatis Rhizoma, Glycyrrhizae Radix Et Rhizoma, etc.		IM	Range: 0.5–5 years	120 (60, 60)	58, 62	49.56 ± 5.67
49	Lou et al., 2008 [44]	Yiqi Sanju formula	Astragali Radix, Coptidis Rhizoma, etc.		WM	N/A	67 (39, 28)	44, 23	T: 52.6 ± 12.8 C: 54.8 ± 11.0
50	Li et al., 2007 [55]	Jianpi Bushen decoction	Astragali Radix, Codonopsis Radix, Atractylodis Macrocephalae Rhizoma, Corni Fructus, Rehmanniae Radix Praeparata, Dioscoreae Rhizoma, etc.		WM	Range: 1–6 years	100 (47, 45)	78, 22	38.5
51	Yang et al., 2007 [56]	Yigan Jiangzhi capsule	Polygoni Multiflori Radix, Curcumae Radix, Ginkgo Folium, Nelumbinis Folium, Salviae Miltiorrhizae Radix Et Rhizoma, Artemisia Scopariae Herba, etc.		WM	T: 2.0 ± 0.5 C: 1.5 ± 0.6	128 (68, 60)	68, 60	T: 45 ± 2 C: 47 ± 4
52	Gu et al., 2007 [24]	Tiaozhi Yanggan decoction	Thorowax Root, Turmeric Root, Red Peony Root, Hawthorn Fruit, Water-Plantain Tuber, Cassia Seed, Giant Knotweed Rhizome, Prepared Rhubarb, Peach Kernel, Red Sage Root, Radish Seed and Tangerine Peel		IM	T: mean = 3 months and 8 years C: mean = 2 months and 6 years	130 (101, 29)	98, 32	T: 47.38 ± 9.50 years C: 43.34 ± 10.54 years
53	Chen et al., 2006 [57]	Qianggan capsule	Astragali Radix, Salviae Miltiorrhizae Radix Et Rhizoma, Angelicae Sinensis Radix, Paeoniae Radix Alba, Curcumae Radix, Codonopsis Radix, Alismatis Rhizoma, Dioscoreae Rhizoma, Crataegi Fructus, Artemisia Scopariae Herba, Glycyrrhizae Radix Et Rhizoma, etc.		IM	N/A	122 (64, 58)	77, 45	T: 42.5 C: 45.8

*T* test group, *C* control group, *H* high dose group, *L* low dose group, *IM* integrative medicine, *WM* Western medicine

74(22, 23,23)*: this study set three group: high dose, low dose and placebo

### Participants

A total of 5997 participants with NAFLD were recruited in the RCTs included in this review. Two trials did not report the number of males and females [29, 30]. The remaining 51 trials reported that 3622 males and 2110 females took part in trials. Among 19 included RCTS, 62 participants were reported as dropouts or withdraw before the intervention was initiated. In terms of allocation, 3165 participants were allocated into the test groups, and 2772 participants were allocated into the control or comparison groups. Out of the 53 RCTs, only 42 reported the mean age of the participants and the standard deviation, 5 trials reported the average age of participants without standard deviation, 3 trials only reported the age range of participants, and 3 trials did not report the age information. Thirty-four trials reported the history of NAFLD in details, and 36 trials reported the participant’s origin. With regards to patient types, 16 trials recruited both outpatients and inpatients as participants, 19 trials recruited outpatients as participants, and one trial recruited inpatients as participants [31]. Four studies did not mention about the trial settings [32–35].

### Diagnosis

Chinese expert consensus and treatment guidelines were the preferred reference for diagnosis standards among the included trials. Fifty-one trials specified the diagnostic criteria including 39 trials selected the relevant standards of Chinese and Western medicine diagnostic, and 12 trials used the Western medicine diagnostic criteria. Detailed information is shown in Table 1. The most common diagnosis criteria reported in the studies were: Guidelines for management of NAFLD (2007 [36], 2010 [37]); Consensus Opinions on the Diagnosis and Treatment of NALFD with TCM and WM [38]. Chinese Medicine Clinical Research of New Drugs Guiding Principles [39]; Expert consensus on TCM diagnosis and treatment of nonalcoholic fatty liver disease [40]. One trial [41] referred the NAFLD diagnostic criteria of the United State [1].

### Intervention

A total of 53 different TCMs preparations were tested in the included trials. The combination of multiple herbs was the main intervention method. All the Chinese medicinal materials from each TCM preparation were summarized as shown in Table 2. The TCMs preparation tested in the included studies might be given in the form of tablets, capsules, decoctions, pills or granules. Most of the TCMs preparations tested were hospitalized-based preparations and no quality standards or quality control of the TCMs being tested was mentioned in any of the RCTs.

At least 86 herbs were used in the included trials for treating NAFLD. The 20 most common Chinese medicinal materials found in the 53 TCMs preparations analyzed in this review included: *Crataegi Fructus*, *Alismatis Rhizoma*, *Atractylodis Macrocephalae Rhizoma*, *Salviae Miltiorrhizae Radix Et Rhizoma*, *Radix Bupleuri*, *Artemisia Scopariae Herba*, *Poria*, *Cassiae Semen*, *Curcumae Radix*, *Astragali Radix*, *Nelumbinis Folium*, *Citri Pericarpium Reticulatae*, *Curcumae Longae Rhizoma*, *Gynostemmatis Herba*, *Angelicae Sinensis Radix*, *Codonopsis Radix*, *Paeoniae Radix Alba*, *Coicis Semen*, *Pinelliae Rhizoma Praeparatum*, *Polygonum Cuspidati Rhizoma Et Radix*. Over 20 trials tested TCMs preparations which consisted of the following 4 Chinese medicinal materials: *Crataegi Fructus*, *Alismatis Rhizoma*, *Atractylodis Macrocephalae Rhizoma*, *Salviae Miltiorrhizae Radix Et Rhizoma*.

### Control and comparison

The control interventions included placebo, conventional medicine, lifestyle intervention, or lifestyle intervention plus conventional drug(s). Thirty-nine trials set a conventional medicine control group, such as polyene phosphatidylcholine capsules (n = 17), silibinin capsules or tablets (n = 4), ursodeoxycholic acid capsules or tablets (n = 3). Four trials used Chinese patent medicine as the intervention in the comparison group. Six trials designed a placebo control group and 32 trials designed a lifestyle intervention, diet or exercise as the comparison group. Detailed information is shown in Table 3.

**Table 3:** Major findings of the RCTs included in the review

No.	Authors, year	Test group		Control group		Duration	Primary outcome		Secondary outcome					
		TCMs	Behavioral interventions	Pharmacological interventions	Behavioral interventions		TCM syndrome score	Adverse reactions	Radiological response	Liver function	Body weight	Blood sugar	Blood lipid	Others
1	Wu et al., 2020 [43]	Jianpi Qingzhi prescription	Diet and exercise	Polyene Phosphatidylcholine capsules	Diet and exercise	12 weeks	Yes	N/A	BU, CT	ALT, AST, GGT	N/A	FBG, FINs, HOMA-IR,	TG, TC	DAO, LPS, d-lactic acid, Claudin-1, Claudin-4
2	Lyv et al., 2020 [47]	Shenge formula	Diet and exercise	N/A	Diet and exercise	6 months	Yes	N/A	N/A	ALT, AST	N/A	N/A	TG, TC	N/A
3	Yang et al., 2019 [59]	Yanggan Jieyu granule	Diet and exercise	Polyene phosphatidylcholine capsules	Diet and exercise	24 weeks	Yes	N/A	CT	ALT, AST, GGT	N/A	N/A	TG, TC, LDL-C	N/A
4	Yang et al., 2019 [97]	Qinjiang Baoling decoction	Diet and exercise	N/A	Diet and exercise	3 months	Yes	N/A	BU, CT	ALT, GGT	BMI	HOMA-IR	TC, HDL-C	N/A
5	Li et al., 2018 [62]	Qutan Huoxue decoction	N/A	Silibinin capsules	N/A	24 weeks	Yes	Yes	CT	ALT, AST, GGT	N/A	N/A	TG, TC	N/A
6	Chen et al., 2017 [98]	Shuangqing granule	Diet and exercise	Xuezhikang capsules	Diet and exercise	12 weeks	Yes	N/A	BU	AST, ALT	N/A	N/A	TG, TC, HDL-C, LDL-C	N/A
7	Hu et al., 2017 [31]	Jianpishugan and Huoxuehuatan formula	Diet and exercise	Polyene Phosphatidylcholine capsules	Diet and exercise	3 months	Yes	Yes	BU	ALT, AST, GGT	BMI	N/A	TG, TC, HDL-C, LDL-C	N/A
8	Jiang et al., 2017 [41]	Dahuang Lidan capsule	Diet and exercise	Polyene Phosphatidylcholine capsules	Diet and exercise	12 weeks	Yes	Yes	BU	ALT, AST, GGT	N/A	N/A	TG, TC, HDL-C, LDL-C	N/A
9	Yang et al.,2017 [53]	Cigu Xiaozhi capsule	Diet and exercise	Polyene Phosphatidylcholine capsules	Diet and exercise	8 weeks	Yes	N/A	BU	ALT, AST, ALP, GGT	BMI	N/A	TG, TC, HDL-C, LDL-C	N/A
10	Jeong et al., 2017 [28]	Test group 1—HL tablet—high dose	N/A	Placebo	N/A	12 weeks	N/A	Yes	N/A	ALT, AST	BMI	HOMA-IR	TG, TC, HDL-C, LDL-C	MRS, free fatty acid
		Test group 2—HL tablet—low dose	N/A	Placebo	N/A	12 weeks	N/A							
11	Sun et al., 2017 [68]	Jianpi Qushi prescription	Diet and exercise	N/A	Diet and exercise	3 months	Yes	N/A	BU	ALT, AST	N/A	FPG, FINs	TG, TC	N/A
12	Niu 2016 [48]	Shugan Jianpi Huatan decoction	Diet and exercise	Atorvastatin calcium tablets	Diet and exercise	3 months	Yes	N/A	N/A	ALT, AST	N/A	FBG, FNIs, HOMA-IR	TG, TC	N/A
13	Zhang et al., 2016 [99]	Yiqi Qizhu decoction	Diet and exercise	Polyene phosphatidylcholine capsules	Diet and exercise	3 months	Yes	Yes	BU	ALT, AST, GGT	N/A	N/A	TG, TC	N/A
14	Zhang et al., 2016 [34]	Jiang Zhi granules	N/A	Ursodeoxycholic acid capsules	N/A	24 weeks	Yes	N/A	BU	N/A	N/A	N/A	N/A	N/A
15	Qi et al., 2016 [63]	Jiangpi Shugan Huoxue Huatan decoction	Diet and exercise	Silibinin capsules	N/A	3 months	Yes	N/A	BU	ALT, AST, GGT	N/A	N/A	TG, TC, HDL-C, LDL-C	N/A
16	Tan et al., 2016 [100]	Shugan Jianpi decoction	Diet and exercise	Pioglitazone	N/A	3 months	Yes	Yes	BU	ALT, AST	BMI, WHR	FBG, FNIs, HOMA-IR	TG, TC	N/A
17	Yu et al., 2016 [69]	Qinggan Jiangzhi decoction	Diet and exercise	Polyene phosphatidylcholine tablet and Dangfei Liganning capsule	Diet and exercise	24 weeks	Yes	N/A	BU	ALT, AST, GGT	BMI, BM	N/A	TG, TC	N/A
18	Yin et al., 2016 [35]	Danxia Zhifang decoction; simulated Zhiganxiao capsule	N/A	Qianggan capsule	N/A	3 months	Yes	N/A	BU, CT	ALT, AST, GGT,TB, DB, TP, ALB, A/G,	N/A	N/A	TG, TC	N/A
19	Zhu 2015 [33]	Qingdan Jiangzhi decoction	Diet and exercise	Tiopronin enteric-coated tablets	Diet and exercise	12 weeks	Yes	N/A	BU	N/A	N/A	N/A	N/A	N/A
20	Li 2015 [64]	Huatan Lishi Huoxue granule combined granule decoction	Diet and exercise	Polyene phosphatidylcholine capsules	Diet and exercise	3 months	Yes	Yes	CT	ALT	N/A	N/A	TG	N/A
21	Chen et al., 2015 [101]	Zaozhu Yinchen decoction	N/A	Silymarin meglumine tablet	N/A	2 months	Yes	Yes	BU	ALT, AST	N/A	N/A	TG, TC	N/A
22	Yu et al., 2015 [52]	Tiaogan Lipi recipe	Diet and exercise	Placebo	Diet and exercise	12 weeks	Yes	Yes	CT	ALT, AST, GGT	N/A	N/A	TG, TC	N/A
23	Li et al., 2015 [58]	Qinghua decoction	Diet and exercise	N/A	Diet and exercise	6 months	Yes	N/A	BU	ALT, AST, GGT, TB	BMI, WHR	N/A	TG, TC	N/A
24	Liu et al., 2014 [49]	Heze lipid-lowering oral liquid	N/A	Polyene phosphatidylcholine capsules	N/A	4 months	Yes	N/A	CT	ALP, AST, GGT, GLP, ALB	BMI	N/A	TG, TC, HDL-C, LDL-C	N/A
25	Liu et al., 2014 [50]	Ruangan Jiangzhi capsule	Diet and exercise	Polyene phosphatidylcholine capsules	Diet and exercise	12 weeks	Yes	Yes	BU	ALT, GGT	N/A	N/A	TG, TC	N/A
26	Li et al., 2014 [51]	Jianpi Shugan recipe	Diet and exercise	Polyene phosphatidylcholine capsules	Diet and exercise	3 months	Yes	Yes	CT	ALT	N/A	N/A	N/A	N/A
27	Zhu et al., 2014 [102]	Zhigan pill	N/A	Placebo	N/A	3 months	Yes	N/A	BU	N/A	N/A	N/A	N/A	N/A
28	Wen et al., 2014 [60]	Jiangan Xiaozhi granule	Diet and exercise	Hedan tablet	Diet and exercise	3 months	Yes	Yes	CT	ALT, AST, GGT	N/A	N/A	TG, TC, HDL-C, LDL-C	N/A
29	Deng, 2013 [103]	Jiangan Sanqing decoction	N/A	Polyene Phosphatidylcholine capsules	N/A	3 months	Yes	N/A	BU	ALT, AST	N/A	N/A	TG, TC	N/A
30	Li et al., 2013 [54]	Jianpi Huatan Zhuyu prescription	N/A	Qianggan capsule	N/A	3 months	Yes	Yes	BU	ALT, AST, GGT	N/A	N/A	TG, TC	N/A
31	Song et al., 2013 [104]	Modified Linggui Zhugan decoction	N/A	Polyene phosphatidylcholine capsules	N/A	3 months	Yes	N/A	CT	ALT, AST	N/A	N/A	TG, TC	N/A
32	Li et al., 2013 [42]	Qushi Huayu decoction	N/A	Polyene phosphatidylcholine capsules	N/A	24 weeks	Yes	Yes	BU	ALT, AST	N/A	N/A	TG, TC, HDL-C, LDL-C	N/A
33	Wong et al., 2013 [26]	*Phyllanthus urinaria*	Diet and exercise	Placebo	Diet and exercise	24 weeks	N/A	Yes	N/A	ALT, AST	N/A	HbA1	HDL-C, LDL-C	N/A
34	Pan et al., 2013 [27]	Jiang Zhi granule	Diet and exercise	Placebo	Diet and exercise	24 weeks	N/A	Yes	CT	N/A	BMI	N/A	TG, TC	N/A
35	Chen et al., 2012 [45]	Jindanwang mixture	Diet	Ursodeoxycholic acid capsules	Diet	24 weeks	Yes	N/A	BU, CT	ALT, AST	N/A	N/A	TG, TC	N/A
36	Zhao et al., 2012 [30]	Huatan Xiezhuo recipe	Diet and exercise	Polyene phosphatidylcholine capsules	Diet and exercise	3 months	Yes	Yes	N/A	ALT	BMI	N/A	TG	N/A
37	Wang et al., 2012 [65]	Yigan Jiedu decoction	Diet and exercise	Silymarin	Diet and exercise	2 months	Yes	N/A	N/A	ALT, AST, GGT	N/A	N/A	TG, TC	N/A
38	Pei et al., 2012 [70]	Qinggan Huatan Huoxue recipe	Diet and exercise	Danning tablet	Diet and exercise	3 months	Yes	Yes	BU	ALT, AST, GGT	BMI	FBG, FNIs, HOMA-IR	TG, TC, HDL-C, LDL-C	N/A
39	Wu 2012 [83]	Jianpi Huatan formula	N/A	Vitamin E	N/A	3 months	N/A	Yes	BU	ALT, AST	N/A	FBG, FNIs, HOMA-IR	TG, TC, HDL-C, LDL-C	N/A
40	Tong et al., 2011 [105]	Jiedu Jianpi Tongluo decoction	N/A	Silibinin capsules	N/A	12 weeks	Yes	Yes	BU	ALT, AST, GGT, TB	N/A	N/A	TG, TC, HDL-C, LDL-C	N/A
41	Zhang et al., 2011 [66]	Kangzhi decoction	N/A	Tiopronin enteric-coated tablets	N/A	6 months	Yes	Yes	BU, CT	ALT, AST, GGT	N/A	N/A	TG, TC	N/A
42	Gu et al., 2011 [61]	Zhigan capsule	Diet and exercise	Polyene phosphatidylcholine capsules	Diet and exercise	3 months	Yes	Yes	BU	ALT, AST, GGT	BMI	FBG	TG, TC, LDL-C	N/A
43	Zhao et al., 2010 [46]	Qiyin tea	N/A	Polyene phosphatidylcholine capsules	N/A	2 months	Yes	N/A	BU	AST, ALP, AST, GGT	N/A	N/A	TG, TC	N/A
44	Ding et al., 2010 [88]	Pingganjian decoction	Diet and exercise	Silymarin, Ultivarietas Oryzae Saltlicae Et Monasci	Diet and exercise	3 months	Yes	Yes	CT	ALT, AST	BMI,WHR	FBG, FNIs, HOMA-IR	TG, TC	N/A
45	Pan et al., 2010 [32]	Tiaozhiji granules	N/A	Hedan tablet	N/A	3 months	Yes	Yes	BU, CT	ALT, AST, GGT	N/A	N/A	TG, TC	N/A
46	Liu et al., 2008 [67]	Qiyin granules	Diet and exercise	Polyene phosphatidylcholine capsules	Diet and exercise	2 months	Yes	N/A	BU	ALT, AST	N/A	N/A	TG, TC	N/A
47	Ji et al., 2008 [25]	Danning tablet	Diet and exercise	Ursodeoxycholic acid tablet	Diet and exercise	24 weeks	Yes	Yes	CT	ALT, AST, GGT	BMI	N/A	TG, TC	N/A
48	Chen et al., 2008 [29]	Xiaoyao San decoction and Danggui Shaoyao San decoction	Diet	Anethol trithione	Diet	3 months	Yes	N/A	BU	ALT, AST	N/A	N/A	TG, TC	N/A
49	Lou et al., 2008 [44]	Yiqi Sanju formula	N/A	Placebo	N/A	3 months	Yes	Yes	CT	ALT, AST	BMI	FBG, FNIs, HOMA-IR	TG, TC, HDL-C, LDL-C	hs-CRP, TNF-α
50	Li et al., 2007 [55]	Jianpi Bushen decoction	N/A	Silibinin	N/A	8 weeks	Yes	Yes	BU	ALT, AST, GGT,TB	N/A	N/A	TG, TC, HDL-C, LDL-C	N/A
51	Yang et al., 2007 [56]	Yigan Jiangzhi capsule	N/A	Dongbao Gantai tablet	N/A	3 months	N/A	N/A	BU, CT	ALT, AST, GGT	N/A	N/A	TG, TC, HDL-C, LDL-C	N/A
52	Gu et al., 2007 [24]	Tiaozhi Yanggan decoction	Diet and exercise	Thiola tablet	Diet and exercise	12 weeks	Yes	Yes	N/A	ALT, AST, GGT	N/A	N/A	TG, TC, HDL-C, LDL-C	N/A
53	Chen et al., 2006 [57]	Qianggan capsule	Diet and exercise	Tiopronin	Diet and exercise	3 months	N/A	N/A	BU	ALT, GGT	N/A	N/A	TG	N/A

No.	Authors, year	Overall findings				
		Efficacy			Safety	
		Overall efficacy rate of test group	Overall efficacy rate of control group	Difference between the 2 groups	Non-serious	Serious
1	Wu et al., 2020 [43]	93.33%	80.00%	Test group (P < 0.05)	N/A	N/A
2	Lyv et al., 2020 [47]	100.00%	48.39%	Test group (P < 0.05)	N/A	N/A
3	Yang et al., 2019 [59]	86.67%	73.33%	Test group (P < 0.01)	N/A	N/A
4	Yang et al., 2019 [97]	64.00%	52.00%	Test group (P < 0.05)	N/A	N/A
5	Li et al., 2018 [62]	84.13%	56.45%	Test group (P < 0.01)	None	None
6	Chen et al., 2017 [98]	89.7%	84.0%	Test group (P < 0.05)	N/A	N/A
7	Hu et al., 2017 [31]	94.28%	77.14%	Test group (P < 0.01)	None	None
8	Jiang et al., 2017 [41]	Specific outcome readings	Specific outcome readings	Test group (P < 0.01)	ADR1	None
9	Yang et al., 2017 [53]	93.02%	68.18%	Test group (P < 0.01)	N/A	N/A
10	Jeong et al., 2017 [28]	Specific outcome readings	Specific outcome readings	High dose group (P = 0.033)	ADR2	None
		Specific outcome readings	Specific outcome readings	Low dose group (P = 0.386)		
11	Sun et al., 2017 [68]	93.33%	61.67%	Test group (P < 0.01)	N/A	N/A
12	Niu 2016 [48]	95.00%	76.60%	Test group (P < 0.05)	N/A	N/A
13	Zhang et al., 2016 [99]	92.31%	78.85%	Test group (P < 0.05)	None	None
14	Zhang et al., 2016 [34]	33.80%	23.39%	Test group (P < 0.05)	N/A	N/A
15	Qi et al., 2016 [63]	93.30%	62.20%	Test group (P < 0.05)	N/A	N/A
16	Tan et al., 2016 [100]	87.23%	71.11%	Test group (P < 0.05)	ADR3	None
17	Yu et al., 2016 [69]	Specific outcome readings	Specific outcome readings	Test group (P < 0.01)	N/A	N/A
18	Yin et al., 2016 [35]	81.63%	62.5%	Test group (P < 0.05)	N/A	N/A
19	Zhu, 2015 [33]	Specific outcome readings	Specific outcome readings	Test group (P < 0.05)	N/A	N/A
20	Li, 2015 [64]	98.10%	80.80%	Test group (P < 0.05)	None	None
21	Chen et al., 2015 [101]	87.50%	73.68%	Test group (P < 0.05)	None	None
22	Yu et al., 2015[52]	Specific outcome readings	Specific outcome readings	Test group (P < 0.05)	None	None
23	Li et al., 2015 [58]	58.33%	30.51%	Test group (P < 0.01)	N/A	N/A
24	Liu et al., 2014 [49]	52.94%	29.09%	Test group (P < 0.05)	N/A	N/A
25	Liu et al., 2014 [50]	91.67%	56.67%	Test group (P < 0.05)	ADR4	None
26	Li et al., 2014 [51]	84.09%	75.00%	Test group (P < 0.05)	ADR5	None
27	Zhu et al., 2014 [102]	85.83%	63.16%	Test group (P < 0.05)	N/A	N/A
28	Wen et al., 2014 [60]	78.40%	55.60%	Test group (P < 0.05)	None	None
29	Deng, 2013 [103]	90.00%	70.00%	Test group (P < 0.05)	N/A	N/A
30	Li et al., 2013 [54]	86.60%	66.60%	Test group (P < 0.05)	None	None
31	Song et al., 2013 [104]	91.70%	69.00%	Test group (P < 0.01)	N/A	N/A
32	Li et al., 2013 [42]	specific outcome readings	specific outcome readings	Test group (P < 0.01)	ADR6	None
33	Wong et al., 2013 [26]	Specific outcome readings	Specific outcome readings	Not siginificant	ADR7	ADR8
34	Pan et al., 2013 [27]	Specific outcome readings	Specific outcome readings	Test group (P < 0.05)	ADR9	None
35	Chen et al., 2012 [45]	94.00%	92.00%	No siginificant differences (P > 0.05)	N/A	N/A
36	Zhao et al., 2012 [30]	Specific outcome readings	Specific outcome readings	Test group (P < 0.01)	None	None
37	Wang et al., 2012 [65]	75.56%	51.11%	Test group (P < 0.01)	N/A	N/A
38	Pei et al., 2012 [70]	87.20%	73.60%	Test group (P < 0.05)	ADR10	None
39	Wu 2012 [83]	83.30%	46.67%	Test group (P < 0.05)	None	None
40	Tong et al., 2011 [105]	86.67%	76.09%	Test group (P < 0.05)	None	None
41	Zhang et al., 2011 [66]	86.90%	67.70%	Test group (P < 0.05)	None	None
42	Gu et al., 2011 [61]	90.00%	72.00%	Test group (P < 0.05)	ADR11	None
43	Zhao et al., 2010 [46]	87.60%	83.90%	No siginificant differences (p = 0.873)	N/A	N/A
44	Ding et al., 2010 [88]	83.33%	45.10%	Test group (P < 0.05)	ADR12	None
45	Pan et al., 2010 [32]	Specific outcome readings	Specific outcome readings	Test group (P < 0.05)	None	None
46	Liu et al., 2008 [67]	Specific outcome readings	Specific outcome readings	Test group (P < 0.05)	N/A	N/A
47	Ji et al., 2008 [25]	Specific outcome readings	Specific outcome readings	Test group (P < 0.05)	ADR13	None
48	Chen et al., 2008 [29]	96.77%	83.87%	Test group (P < 0.05)	N/A	N/A
49	Lou et al., 2008 [44]	94.87%	32.14%	Test group (P < 0.01)	ADR14	None
50	Li et al., 2007 [55]	87.23%	71.11%	Test group (P < 0.05)	None	None
51	Yang et al., 2007 [56]	88.20%	71.70%	Test group (P < 0.05)	N/A	N/A
52	Gu et al., 2007 [24]	81.19%	68.97%	Test group (P < 0.05)	ADR15	None
53	Chen et al., 2006 [57]	79.69%	62.07%	Test group (P < 0.05)	N/A	N/A

*ALB* albumin, *ALP* alkaline phosphatases, *ALT *aminotransferase, *AST* aspartate aminotransferase, *A/G* albumin/globulin ratio, *BU* B-ultrasound, *CT* computed tomography, *DAO* diamine oxidase, *DB* direct bilirubin, *FBG* fasting blood glucose, *FINS* fasting plasma insulin, *GGT* gamma-glutamyl-transpeptidase, *GLB* globulin, *HDL* high density lipoprotein-cholesterol, *HFC* hepatic fat content, *HOMA-IR* homeostasis model assessment-estimated insulin resistance, *hs-CRP* high sensitivity C-reactive protein, *L/S* ratio liver to spleen ratio, *LDL* low density lipoprotein-cholesterol, *MRS* magnetic resonance spectroscopy, *TB* total bilirubin, *TC* total cholesterol, *TG* triglyceride, *TP* total serum protein, *TNF-α* tumor necrosis factor-α, *VLDL* very low density lipoprotein, *WHR* waist/hip ratio

ADR1: one patient in the control group had a mild prothrombin time

ADR2: high dose group: abdominal pain upper(1 patient); low dose group: dizziness(1 patient); Palcebo group: nausea (1 patient)

ADR3: one case in the control group had mild edema of both lower limbs

ADR4: 4 patients in the test group had different degrees of diarrhea

ADR5: 1 case in test group; 3 cases in control group(not specified)

ADR6: in the test group, 3 cases developed nausea and 2 cases of gastric cavity discomfort; in the control group, 2 cases of gastric cavity discomfort

ADR7: dyspepsia, diarrhea, per-rectal bleeding, chest pain, cough, headache, blurred vision, toothache, gum bleeding, flu-like symptoms

ADR8: hospitalization occurred in two patients in the Phyllanthus group (back pain, stroke) and one patient in the placebo group (acute appendicitis)

ADR9: 16 patients with 19 cases of adverse events were reported in the test group, and 14 patients with 19 cases were reported tn the control group (no mention of the mild adverse name). 1 case exhaustion/dizziness and 1 case sloppy stool were related the study

ADR10: 3 cases in the test group had loose stools; In the control group, 2 cases had nausea and 5 cases had loose stools

ADR11: 5 patients in the test group had different degrees of diarrhea symptoms

ADR12: in the test group, 3 patients had mild diarrhea, gastrointestinal discomfort and reduced appetite, 1 case of hypertension; In the control group 2 cases of hypertension

ADR13: in test group, most of pat ients showed diarrhea. There was one patient with skin rash and three patients with nausea

ADR14: in the test group, 3 patients developed mild diarrhea, recovered and decreased appetite

ADR15: 21 cases of diarrhea, 22 of gastric discomfort or light pain, 15 of abdominal discomfort or dull pain, and 11 cases of nausea

All the comparisons were:

- TCMs versus placebo (2 trials);
- TCMs plus lifestyle intervention versus placebo plus lifestyle intervention (4 trials);
- TCMs plus lifestyle intervention versus lifestyle intervention (4 trials);
- TCMs versus conventional medicine (19 trials);
- TCMs plus lifestyle intervention versus conventional drug plus lifestyle intervention (18 trials);
- TCMs plus lifestyle intervention (only diet) versus conventional drug plus lifestyle intervention (only diet) (2 trials);
- TCMs versus TCMs (1 trial);
- TCMs plus lifestyle intervention versus TCMs plus lifestyle intervention (3 trials).

### The intervention durations

The duration of the intervention among the included trials ranged between 8 weeks and 6 months. In 34 trials, 12 weeks or 3 months was set as intervention duration; in 12 trials, 24 weeks or 6 months was set as the intervention duration; in 6 trials, 8 weeks or 2 months were the intervention duration; and 1 trial set 4 months as intervention duration.

### Outcomes

As shown in Table 3, the reported outcomes of the 53 trials included changes in the TCM syndrome score and any reports of adverse reactions, along with B-ultrasound findings, computed tomography (CT) scan findings, body weight (BMI, BM), related biochemical response measures of 1iver function (ALT, AST, ALP, GGT, TB, TP, ALB, A/G, GLB, DB), related biochemical response measures of blood sugar (FBG, FNIs, HOMA-IR, Hb1A, MRS), related biochemical response measures of blood lipids (TG, TC,HDL-C, LDL-C, CHOL), and others (DAO, lipopolysaccharide, d-lactic acid, Claudin-1, Claudin-4, hs-CRP, TNF-α).

All trials measured the outcomes at the end of the intervention duration, and no RCTs reported any follow-up data of the outcomes after the interventions ended. The most commonly measured outcomes included related biochemical response measures of 1iver function (n = 49), blood lipids (n = 49), TCM syndrome score (n = 47) and B-ultrasound findings and computed tomography (CT) scan findings (n = 46). Blood sugar levels (n = 12) and body weight (n = 15) were less frequently used as part of the outcome measurements. Most of the RCTs (n = 41) compared the overall efficacy of interventions in the test groups and the control/comparison groups whereas 12 RCTs compared the 2 groups in terms of the each of the specific outcome measures in each study. All but 3 RCTs showed positive effects of TCMs on the outcomes measured in the test groups compared to the control/comparison groups with statistical differences.

All the 28 RCTs set out to measure the safety of the TCMs were the only RCTs which reported the data of adverse events experienced by the participants. Fourteen of these 28 RCTs did not identify any adverse events associated with the interventions used in the studies. Among the remaining 14 RCTs which reported adverse events, 13 RCTs reported only non-serious adverse events and 1 RCT reported both serious and non-serious adverse events in which hospitalization occurred in two participants from the test group receiving phyllanthus due to back pain and stroke and one participant in the placebo group had acute appendicitis [26]. Non-serious adverse effects mainly included gastrointestinal discomfort (such as diarrhea, gastric discomfort or light pain, nausea, diarrhea, decreased appetite) and other mild complaints about cough, headache, blurred vision, dizziness, toothache, gum bleeding, and flu-like symptoms. Thirteen trials reported that the adverse reactions were alleviated by symptomatic treatment with no influence on the trials. None of the trials reported any death from any cause.

All the comparisons were:

- TCMs versus placebo (2 trials);

Further information about the reporting of each outcome measurements is provided in the following:

- Radiological response (BU, CT)
  - Seven trials conducted the B-ultrasound and CT to evaluate the efficacy of treatment on NFALD. 12 trials only conducted the CT results before and after treatment, and 27 trials only conducted B-ultrasound results.

- Liver function
  - Out of the 47 trials which tested the related biochemical response measures of liver function, 19 trials reported the changes in AST, ALT, GGT, 17 trials reported ALT and AST change, 4 trials reported ALT, AST, ALP and GGT change, and 4 trials also reported TB. One trial also reported the MRS and free fatty acid to assess of hepatic fat content [28].

- Blood sugar
  - Out of the 12 trials that reported the results of the related biochemical response measures of blood sugar, 7 trials reported FBG, FNIs, HOMA-IR results, and 1 trial [42] reported the Hb1A results.

- Blood lipids
  - Among the 49 trials that reported the results of blood lipids. 43 trials reported the TG and TC results, of which 16 trials reported the TG, TC, HDL-C and LDL-C results.

- Others (DAO, lipopolysaccharide, d-lactic acid, Claudin-1, Claudin-4, hs-CRP, TNF-α).
  - One trial [43] reported the results of DAO, lipopolysaccharide, d-lactic acid, Claudin-1, Claudin-4, and one trial [44] reported the hs-CRP, TNF-α to test the liver inflammation.

### The overall efficacy

The final result of 50 trials reported that the test group was more effective than the control group, of which 37 trials showed statistical difference at a two-sided P-value of less than 0.05, while 13 trials showed statistical differences taken p < 0.01. A total of 41 trials reported the overall efficacy rates, the effective rate of the test groups ranged from 33.80 to 100%, of which 16 trials reported the overall effective over 90%, and only 1 [34] trial below 50%. The reported overall efficacy rate of control group ranged from 23.39 to 92.00%, of which only 1 [45] trial reported the overall efficacy rate of over 90%, and 7 trials below 50%.

Compared with Ursodeoxycholic Acid Capsules, one trial found that the Jindanwang Mixture was equally effective in treating NAFLD, with the overall efficacy rate 94% vs. 92% in (p > 0.05) [45]. One trial reported that Qiyin Tea and Polyene Phosphatidylcholine Capsules were both equally effective in managing NAFLD [46]. On the other hand, Phyllanthus was shown to be superior to placebo in improving NAFLD (p = 0.873) in one trial [26]. Many of the TCMs were shown to have beneficial effects on the TCM syndrome score, liver function, body lipid profile, blood sugar level and body weight. For instance, a 6-month treatment with Shenge Formula with behavioral interventions, when compared to behavioral interventions only, was more effective in improving liver function and blood lipid profile, yielding a 100% efficacy rate in the test group versus 48.39% in the comparison group [47]. A 3-month treatment with Shugan Jianpi Huatan Decoction with behavioral interventions, when compared to the use of atorvastatin and behavioral interventions, was more effective in improving liver function, blood sugar level and blood lipid profile [48]. A 4-month treatment with Heze lipid lowing oral liquid decoction along was more effective than polyene phosphatidylcholine [49] in improving the liver function, body weight and body lipid profile. All 3 TCMs also appeared to be effective in improving the TCM syndrome score without inducing any risks of adverse effects.

### CONSORT-CHM

The summary of the CONSORT-CHM quality assessment results of the 53 RCTs included in this review is shown in Table 4. None of the RCTs fully met all the CONSORT-CHM criteria. The most common reasons for non-compliance in descending order were: a lack of “Other information” (which included information about funding sources, where the full trial protocol could be assessed, and the registration number and the name of trial registry); a lack of discussion about trial limitations; incomplete results due to a lack of information about all the important harms and unintended effects in each group and a lack of ancillary analyses; and incomplete information about the trial methods (which included description about participant flow, blinding, allocation methods and implementation).

**Table 4:** Evaluation of included trial studies using the CONSORT-CHM statement

Number	Authors, year	Title/abstract	Background	Objectives	Trial design	Participants	Interventions	Outcomes	Sample size	Randomization	Allocation	Implementation	Blinding	Statistical methods
1	Wu et al., 2020	△	◯	◯	△	◯	◯	◯	△	△	△	×	×	◯
2	Lyv et al., 2020	△	◯	◯	△	◯	◯	◯	△	△	△	×	×	◯
3	Yang et al., 2019	△	△	×	△	△	△	◯	△	△	△	×	×	◯
4	Yang et al., 2019	△	△	×	△	◯	◯	◯	△	△	×	×	×	◯
5	Li et al., 2018	△	△	◯	△	◯	△	◯	△	△	△	×	×	◯
6	Chen 2017	△	△	×	△	△	△	△	△	△	×	△	×	◯
7	Hu et al., 2017	△	×	△	△	◯	△	△	△	△	×	△	×	◯
8	Jiang et al., 2017	△	△	×	△	◯	△	△	△	△	×	△	×	◯
9	Yang et al., 2017	△	△	△	△	△	△	◯	△	△	△	×	×	◯
10	Jeong et al., 2017	◯	◯	◯	◯	△	△	◯	△	△	△	×	×	◯
11	Sun et al., 2017	△	×	×	△	◯	△	△	△	△	×	△	×	◯
12	Niu 2016	△	◯	△	△	◯	△	◯	△	△	×	×	×	△
13	Zhang et al., 2016	△	◯	◯	△	◯	◯	◯	△	△	△	△	×	◯
14	Zhang et al., 2016	△	×	×	△	◯	△	△	△	△	×	△	×	△
15	Qi et al., 2016	△	△	◯	△	◯	◯	◯	△	△	×	×	×	◯
16	Tan et al., 2016	△	△	×	△	◯	◯	◯	△	△	×	×	×	◯
17	Yu et al., 2016	△	△	×	◯	◯	△	△	△	△	×	△	×	◯
18	Yin et al., 2016	△	△	△	◯	◯	△	◯	△	△	×	×	△	◯
19	Zhu 2015	△	△	×	△	◯	◯	◯	△	△	△	×	×	◯
20	Li 2015	△	△	×	×	△	△	◯	△	△	×	×	×	◯
21	Chen et al., 2015	△	△	△	△	◯	◯	◯	△	△	△	×	△	◯
22	Yu et al.,2015	◯	◯	◯	△	◯	◯	◯	△	△	×	×	×	△
23	Li 2015	△	△	△	△	◯	△	◯	△	△	△	×	×	◯
24	Liu et al., 2014	△	◯	◯	◯	◯	◯	◯	△	△	×	×	×	◯
25	Liu et al., 2014	△	×	△	△	△	△	◯	△	△	×	×	×	◯
26	Li et al., 2014	◯	◯	◯	◯	◯	◯	◯	△	△	△	◯	△	◯
27	Zhu et al., 2014	△	△	◯	◯	◯	△	◯	△	△	×	△	×	◯
28	Wen et al., 2014	△	△	×	△	△	◯	◯	△	△	×	×	×	◯
29	Deng, 2013	△	△	◯	◯	◯	◯	◯	△	△	×	×	×	◯
30	Li et al., 2013	△	△	△	△	△	△	△	△	△	×	×	×	◯
31	Song et al., 2013	△	△	×	△	◯	◯	◯	△	△	×	×	×	◯
32	Li et al., 2013	△	△	◯	△	◯	△	△	△	△	×	△	×	◯
33	Wong et al., 2013	◯	◯	△	◯	◯	△	◯	△	△	△	◯	△	◯
34	Pan et al., 2013	◯	◯	◯	◯	◯	◯	◯	△	△	△	×	×	◯
35	Chen et al., 2012	△	△	×	△	◯	△	△	△	△	×	△	×	◯
36	Zhao et al., 2012	△	△	△	◯	△	△	△	△	◯	△	◯	△	△
37	Wang et al., 2012	△	△	×	△	◯	△	△	△	△	×	△	×	◯
38	Pei et al.,2012	△	◯	×	◯	◯	◯	◯	△	◯	◯	×	×	◯
39	Wu et al., 2012	△	×	×	△	△	△	△	△	△	×	×	×	◯
40	Tong et al., 2011	△	△	◯	△	◯	△	△	△	△	×	△	×	◯
41	Zhang et al., 2011	△	△	×	△	△	△	△	△	△	×	△	×	◯
42	Gu et al., 2011	△	△	◯	△	△	△	△	△	△	×	△	×	◯
43	Zhao et al., 2010	△	△	×	△	◯	△	◯	△	△	×	△	×	◯
44	Ding et al., 2010	△	△	◯	△	◯	△	△	△	△	×	△	×	◯
45	Pan et al., 2010	△	△	△	△	△	△	△	△	△	×	△	×	◯
46	Liu et al.,2008	△	△	△	△	◯	△	◯	△	◯	△	△	△	◯
47	Ji et al., 2008	◯	△	◯	◯	◯	△	△	△	◯	×	△	×	◯
48	Chen et al., 2008	△	△	◯	△	◯	△	◯	△	△	×	△	×	△
49	Lou et al., 2008	△	◯	△	◯	◯	△	△	△	◯	△	△	△	△
50	Li et al., 2007	△	◯	◯	△	△	△	◯	△	△	×	△	×	◯
51	Yang et al., 2007	△	×	×	△	△	△	△	△	△	×	△	×	◯
52	Gu et al., 2007	△	◯	△	◯	◯	△	△	△	◯	△	×	×	△
53	Chen et al., 2006	△	△	△	△	△	△	△	△	△	×	△	×	△

Number	Authors, year	Participant flow	Recruitment	Baseline data	Numbers analysed	Outcomes	Ancillary analysis	Harms	Limitations	Generalizability	Interpretation	Registration	Protocol	Funding
1	Wu et al., 2020	×	△	◯	◯	△	×	×	×	◯	◯	×	×	◯
2	Lyv et al., 2020	×	△	△	◯	△	×	×	×	◯	◯	×	×	◯
3	Yang et al., 2019	×	△	◯	◯	△	×	×	◯	△	△	×	×	◯
4	Yang et al., 2019	×	△	◯	◯	△	×	×	×	◯	◯	×	×	◯
5	Li et al., 2018	×	△	△	◯	△	×	◯	◯	△	△	×	×	◯
6	Chen 2017	×	△	◯	△	△	×	×	×	△	◯	×	×	◯
7	Hu et al., 2017	×	◯	◯	◯	△	×	◯	×	◯	◯	×	×	◯
8	Jiang et al., 2017	×	◯	◯	◯	△	×	◯	×	△	◯	×	×	◯
9	Yang et al., 2017	×	△	△	△	△	×	×	×	△	△	×	×	×
10	Jeong et al., 2017	◯	×	◯	◯	△	×	◯	△	△	△	◯	◯	×
11	Sun et al., 2017	×	◯	◯	△	△	×	×	×	△	◯	×	×	◯
12	Niu 2016	×	△	◯	◯	△	×	×	×	△	△	△	×	×
13	Zhang et al., 2016	×	△	×	◯	△	×	◯	×	△	△	×	×	◯
14	Zhang et al., 2016	×	×	◯	◯	△	×	×	×	△	△	×	×	◯
15	Qi et al., 2016	×	△	◯	◯	△	×	×	×	△	△	×	×	×
16	Tan et al., 2016	×	△	◯	◯	△	×	◯	×	△	△	×	×	×
17	Yu et al., 2016	×	△	◯	◯	△	×	×	×	△	△	×	×	◯
18	Yin et al., 2016	×	△	◯	△	△	×	×	×	△	△	×	×	◯
19	Zhu 2015	×	△	◯	◯	△	×	×	×	△	△	×	×	×
20	Li 2015	×	◯	◯	◯	△	×	◯	×	△	△	◯	×	×
21	Chen et al., 2015	×	△	◯	◯	△	×	◯	×	△	△	×	×	◯
22	Yu et al.,2015	×	△	◯	△	◯	×	◯	×	△	△	×	×	◯
23	Li 2015	×	△	△	◯	△	×	×	×	△	△	×	×	◯
24	Liu et al., 2014	×	△	×	◯	△	×	×	×	△	△	×	×	×
25	Liu et al., 2014	×	△	△	◯	△	×	△	×	△	△	×	×	◯
26	Li et al., 2014	×	△	◯	◯	△	×	◯	×	△	△	◯	×	×
27	Zhu et al., 2014	×	◯	△	◯	△	×	×	×	△	△	×	×	×
28	Wen et al., 2014	×	△	△	◯	△	×	◯	×	△	△	×	×	×
29	Deng, 2013	×	△	×	◯	△	×	×	×	△	△	×	×	×
30	Li et al., 2013	×	△	△	◯	△	×	◯	×	△	△	×	×	×
31	Song et al., 2013	×	△	◯	◯	△	×	×	×	△	△	×	×	×
32	Li et al., 2013	△	△	△	◯	△	×	◯	×	◯	△	×	×	×
33	Wong et al., 2013	◯	△	◯	◯	◯	×	◯	◯	△	△	◯	◯	×
34	Pan et al., 2013	◯	△	◯	◯	◯	△	◯	◯	△	△	◯	×	×
35	Chen et al., 2012	×	△	△	◯	△	×	×	×	◯	△	×	×	◯
36	Zhao et al., 2012	×	△	△	◯	△	△	◯	×	△	△	△	×	◯
37	Wang et al., 2012	×	△	△	◯	△	×	×	×	◯	◯	×	×	×
38	Pei et al.,2012	×	△	◯	◯	◯	×	◯	×	△	△	×	×	◯
39	Wu et al., 2012	◯	◯	△	△	△	×	◯	×	◯	△	×	×	◯
40	Tong et al., 2011	×	△	△	◯	△	×	◯	×	◯	△	×	×	×
41	Zhang et al., 2011	×	◯	◯	◯	△	×	◯	×	◯	△	×	×	×
42	Gu et al., 2011	×	◯	◯	◯	△	×	◯	×	◯	△	×	×	×
43	Zhao et al., 2010	×	△	×	◯	△	×	×	×	◯	◯	×	×	×
44	Ding et al., 2010	×	×	◯	◯	△	×	◯	×	◯	△	×	×	×
45	Pan et al., 2010	×	△	×	◯	△	×	◯	×	◯	△	×	×	×
46	Liu et al.,2008	×	◯	△	◯	△	×	×	×	◯	◯	×	×	×
47	Ji et al., 2008	×	△	◯	◯	△	×	△	×	◯	◯	×	×	◯
48	Chen et al., 2008	×	◯	◯	◯	△	×	×	×	◯	◯	×	×	×
49	Lou et al., 2008	×	◯	◯	◯	△	△	◯	×	△	△	△	×	×
50	Li et al., 2007	×	◯	×	◯	△	×	◯	×	△	◯	×	×	×
51	Yang et al., 2007	×	◯	△	◯	△	×	×	×	△	△	×	×	×
52	Gu et al., 2007	△	△	×	◯	△	×	◯	×	△	◯	◯	×	×
53	Chen et al., 2006	×	△	△	◯	△	△	×	×	△	△	△	×	×

All 53 trials reported randomization, but only 6 of them fully reported the method of generating random sequences, including 4 trials used random number tables and 2 RCTs used the central randomized method. Only 3 trials specified the implementation of clinical trials, including random methods, recruitment of participants, and intervention process. None of the studies fully described how to conduct blinding. Seven trials mentioned that blinding method was used, but there was a lack of detailed descriptions of blinding personnel and implementation methods. Forty-six trials did not mention the blinding procedures at all. Only 4 trials fully reported the primary outcome, the secondary outcome, and the outcome of the relevant indicators stated in the method. The remaining 46 trials did not report all the outcomes that the study design had set to measure. Among the 14 trials reported the adverse reaction, 2 trials did not specify information of the cases number of test group or control group. A total of 25 trials did not include adverse effects as one of the outcome measurements.

### Risk of bias

**Fig. 2:**
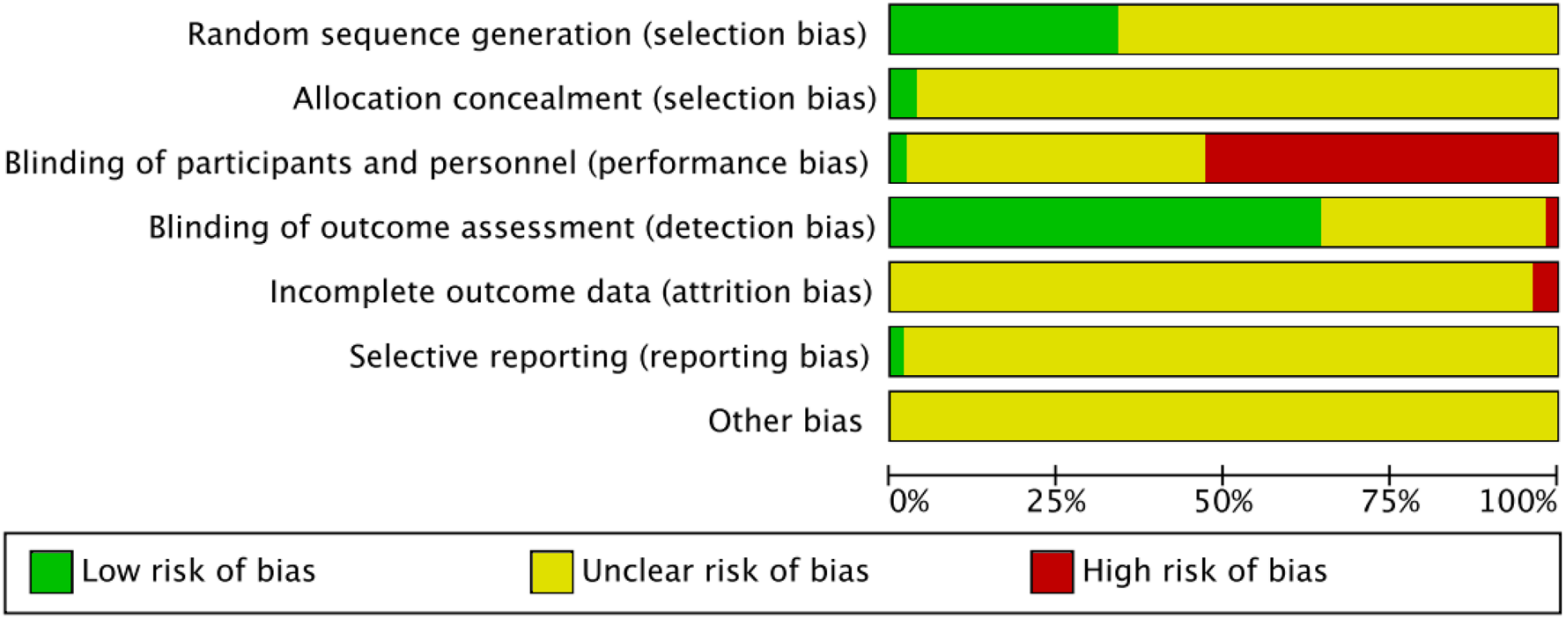
Risk of bias graph: the judgements of the review authors about each domain of the risk of bias presented as percentages across all included studies

**Fig. 3:**
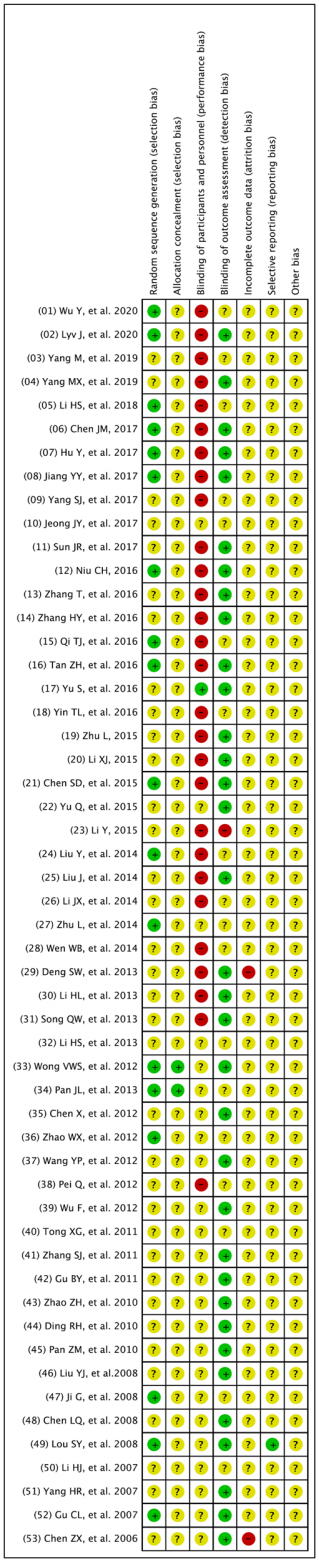
Risk of bias summary: the judgements of the review authors about each risk of bias item presented for each included trial

Most of the trials provided limited information about study design and methodology. Five multicentre randomized clinical trials were identified [27, 30, 49–52]. While most of the trials (n = 42) had clearly specified both inclusion criteria and exclusion criteria in the trial design, 5 trials did not prespecify them [53–57]. Three trials had not pre-specified inclusion criteria [32, 50, 58] and 3 trials had not pre-specified exclusion criteria [59–61]. The authors’ judgements about each domain of risk of bias are presented as percentages across all included trials as shown in Fig. 2, and the judgment about each risk of bias for each included trial is shown in Fig. 3. None of the trials was accessed to have low risk of bias for all domains. All included trials were accessed at unclear or high risk of bias in one or more domains.

Further information about the judgement of the 6 domains of the risks of bias is provided in the following:

- Randomization
  - We assessed 18 trials at low of bias due to adequate reporting and application of random sequence generation, the remainder were assessed at unclear risk of bias.

- Allocation (sequence generation and allocation concealment)
  - Two trials reported detailed information on allocation concealment methods and were regarded as adequate [26, 27], and 51 trials were assessed at unclear risk of bias due to not providing the specific methods of allocation concealment.
    Blinding
  - Two trials claimed that they respectively used single-blinding or double-blinding but they did not report who was blinded [28, 58]. Two trial reported that it used blindness-method evaluation [51, 52]. One trial reported specifically who were blinded to the treatment assignment [26]. The remaining trials did not mention blinding.
    Incomplete outcome data
  - Three trials specified the numbers and reasons of withdrawal and loss to follow-up and were assessed as trials with a low risk of bias [26, 47, 52]. One trial gave unclear information on withdrawals and loss to follow-up [58]. 18 trials reported participants lost to follow-up without providing the reasons for the loss and were assessed as trials with unclear risk of bias [28, 30–32, 43–45, 50, 55, 62–70]. The remaining trials reported that no participants were lost to follow-up during the trial.
    Selective reporting
  - Only 2 trials specified outcome measures were not reported [60, 61] and the remaining trials were assessed as having high risk of reporting bias. All 53 trials were considered to have unclear risks of bias due to lack of adequate information.
    Other potential sources of bias
  - All the trials did not report the sample size calculations and were assessed as not free of other potential sources of bias.

## Discussion

This systematic review identified RCTs and evaluate clinical evidence for the use of TCMs on treating NAFLD. Fifty-three RCTs involving 5997 participants were included. The mean sample size was 113 participants (range 60 to 221 participants) per trial. Each included RCT tested different TCMs giving a total of 53 TCMs identified in this study. The most common Chinese medicinal materials found in the investigated TCMs were *Crataegi Fructus* (山楂), *Alismatis Rhizoma* (澤瀉), *Atractylodis Macrocephalae Rhizoma* (白術) and *Salviae Miltiorrhizae Radix Et Rhizoma* (丹參). TCMs (with or without behavioral interventions) have been shown to be more effective statistically than the control, placebo, pharmacological interventions (with or without behavioral interventions) (P < 0.05 or P < 0.01) as reported in 50 RCTs. The present systematic review also suggests that TCMs such as Shenge formula, Shugan Jianpi Huatan decoction and Heze lipid lowing oral liquid decoction might have positive effects on NAFLD by improving TCM syndrome scores, liver function, and body lipid profile. A range of non-serious, reversible adverse effects associated with the use of TCMs was also reported.

Nevertheless, the evidence reported in the included RCTs is not sufficient to recommend any of the investigated TCMs for the treatment of NAFLD due to the questionable quality of RCTs, the risk of bias and the lack of homogeneous data. None of the included RCTs was in full compliance with the CONSORT-CHM guideline. The risks of bias were either identified or could not be ruled out due to incomplete information. The heterogeneity in the methodology across all the trials meant that a meta-analysis was not possible, and thus further recommendation about a common or predominant medicine for NAFLD could not be made. Improvement in the RCT protocol, the use of a larger sample size, a setting of multicenter and a more focused approach in selecting TCMs for testing in the future might allow more reliable evidence to be developed effectively to support the role of TCMs in NAFLD treatment. Further analysis of the RCTs included in this review identified common limitations and provided an insight about priority areas of improvement for RCTs testing TCMs as discussed in the following.

Firstly, the reporting quality of the 53 RCTs was generally poor. None of the trials completely fulfilled the criteria of the CONSORT-CHM statement. The evaluation results of the Cochrane risk-of-bias tool were also concerning in light of the multiple risks of bias associated with the randomization sequence generation and blinding. Although all the trials reported the study protocol was designed according to recommended standards, only 3 RCTs fully reported the randomization sequence generation in detail, and none of the 53 trials conducted a blinding process in full compliance. Blinding is important in minimizing bias and maximizing the validity of the study results. As a review of 3159 RCTs previously reported, compared with the RCTs using blinding, the RCTs not using blinding yielded 17% larger estimates of treatment effects and in trials with subjective outcomes, the effect estimates could be exaggerated by 25% [71]. Nevertheless, researchers seemed to have failed to make improvement in the study design and implementation and the issues about blinding procedures in RCTs testing TCMs or other herbal medicines were repeatedly reported and were once again reiterated in this study [72–74].

Secondly, most of the RCTs included in this review measured the TCM syndrome score to estimate the efficacy of the investigated TCMs. The score system is determined based on the assessment of TCM symptoms and signs, and is one of the most important indexes for evaluating the effectiveness of a TCM in the treatment of a disease [75]. Previously, this scoring system has been repeatedly used as the primary endpoints to examine the efficacy of TCMs for treatment of various conditions [76–79]. The scoring items mainly involved dry mouth, bitter eyes, dry eyes, bleeding gums, insomnia and dreams, abdominal distension, loss of appetite, fatigue, loss of appetite, hypochondriac pain, waist and knee pain, urine and bowel, etc. [39]. The scores were determined by TCM practitioners based on the assessment of clinical symptom and sign with a degree of clinical manifestation as observed. However, it was not reported in details in the included RCTs how the repeatability and reliability of TCM syndrome score assessment results were ensured. For this, the assessment should be repeated by at least 1 TCM practitioners separately in the same condition to verify the final scoring results [80]. The reporting of the TCM syndrome score results should be made specific to the syndromes assessed to rule out any concerns of incomplete outcomes data or selective reporting bias.

Thirdly, the limited sample size as reported in some of the included RCTs was another cause of concern. While most of the included RCTs recruited nearly 100 participants and some having over 200 participants, 19 trials (35.19%) reported less than 100 participants in the clinical studies. Insufficient participant’s number may result in the inability to detect a precise effect and affect the statistic bias and the quality of trial, possibly leading to a lack of statistical ability to properly estimate the effect of treatment and overestimating the risk of intervention benefit. A sample size calculation involves determining the minimum number of participants needed to detect a treatment effect that is clinically relevant [81]. As depicted in the CONSORT-CHM guidelines, there was a special emphasis on sample size, with recommendations to explain how the sample size was determined which allows a high probability of detecting a statistically significant, clinically relevant difference if one exists [16]. However, most sample size calculation parameters in RCTs testing TCMs may remain poorly understood making it difficult to estimate the sample size needed. One potential solution was ‘‘sample size samba’’ or ‘‘delta inflation’’, whereby investigators commonly start with the number of available participants and adjust their estimates of the sample size calculation assumptions to justify their sample size [82]. Importantly, the sample size calculation should still be adequately conducted and fully reported to ensure and demonstrate methodological quality.

The age and sex of participants in the included trials were representative factors of people with NAFLD. Nearly all the included RCTs recruited participants in China and only 1 RCT was conducted abroad [28]. This may have had an impact on the generalizability of the evidence and the applicability of the interventions to other populations. No data longer than 6 months on any of the post-treatment follow-up or single-trial outcomes were reported in the included trials. Therefore, the long-term safety and effects of the tested TCMs remained unknown warranting further research in the future. On the other hand, the age attribute of the participants in most of the included RCTs (the range of mean age across the RCTs being 36.9 to 57.3 years old) appeared to be consistent with the common onset age of NAFLD. Only 1 RCT [83] reported the effects of TCMs on children patients with NAFLD. With the increase in obese children all over the world, NAFLD has been considered an important liver disease in children illness [84, 85]. Obese children with clinical or biochemical hepatic abnormalities are prone to suffer from NAFLD [86] and can develop NASH at the age of 4 [83]. The landscape of therapeutic developments in pediatric NAFLD is expected to expand. For this, future RCTs should take into consideration the need for evidence of treatment in children NAFLD. The lack of information about the quality standards and quality control of the investigated TCMs might inevitably cause reasonable doubts about the safety of TCMs used by the participants. The reporting of future RCTs should provide supporting information about standardization including composition, quality control, detailed dose regimen, and manufacturing process [12].

Many crude extracts from medicinal plants have significant anti-NAFLD effects. For instance, berberine, which was isolated from the Chinese medicinal material *Coptidis Rhizoma* and widely used to treat diarrhea and other inflammatory diseases in China [87], was the component of Pinggan jian Decoction [88] and Yiqi Sanju Formula [44] analyzed in this review. Recent studies have proved a new therapeutic function of berberine in metabolic disorders, including obesity and diabetes [89, 90]. Berberine can be used as a cholesterol lowering drug, through a unique mechanism distinct from statins[91]. These studies suggested a potential therapeutic activity of berberine for NAFLD. Besides 53 TCM formulas in these systematic review, many TCMs are reported to have significant anti-NAFLD effects. One famous TCM, Yinchenhao Decoction, first recorded in the “Shen Nong’s Herbal Classic”, has been used in treatment of gallbladder and liver diseases for centuries. It can reduce the accumulation of hepatic fat, enhance adiponectin secretion, increase endothelial progenitor cell proliferation, and increase PPAR-γ expression, which is probably responsible for the therapeutic effect of YCHD on NAFLD [92, 93]. Another well-known TCM formula, Qushi Huayu decoction can effectively reverse elevated levels of free fatty acid and total triglycerides (TG), and also can improve hepatic steatosis and inflammation [10]. While treatment options for NAFLD remain limited, the role of TCMs as an indispensable resource for the development of liver protection drugs should be appreciated and the continuous effort in developing quality evidence about the efficacy and safety is pivotal.

Apart from efficacy, evaluation of the safety of TCMs in the management of NAFLD was another primary goal of this review. However, no clear conclusions can be made about the safety of TCMs due to inadequate reporting on adverse reactions in the included trials. If the studies were to be assessed for causality, it would clearly show that adverse events caused by TCMs are relatively infrequent [94]. TCMs when compared with placebo, rarely carry a higher risk of adverse effects according to the results of RCTs [95]. However, an increased incidence of rare adverse events or events with significant latency cannot reliably detected in RCTs [96]. Hence, reliable and comprehensive assessment on TCMs safety should be informed based on an integrative assessment of the totality of the available clinical data (RCTs, case reports, spontaneous reporting schemes and post‐marketing surveillance studies).

There were several limitations in our study. Although only RCTs were included in our study, the quality of the RCT design was not high. Together with the vastly different TCMs investigated and the lack of homogeneity in the study design across the included RCTs, it was difficult to draw a clear conclusion about the efficacy and safety of TCMs for NAFLD. Moreover, the evaluation of the TCMs efficacy and safety was based on the quality assessment in this study which was in turn based on how the original study was reported. The judgment about the TCMs efficacy and safety, therefore, may be subject to the influence of the quality of reporting. The CONSORT-CHM extensions adopted as the evaluation framework in this review were designed to improve the completeness and transparency of reporting of interventions in controlled trials of Chinese herbal medicines. However, few researchers had adopted the recommendations fully. By employing the CONSORT-CHM guidelines as an assessment tool and revealing the shortfalls in the current RCT design and reporting, it is anticipated that the quality of TCMs RCT research would be improved through improved awareness, recognition and adoption of the CONSORT-CHM guidelines.

## Conclusion

Based on this review, the efficacy of TCMs in NAFLD management appears to be promising and the risks associated with the investigated TCMs were reportedly minimal. However, no conclusion can be made so far due to the concerns over the quality of RCTs and the possible risks of bias. Improvement in the RCT protocol, the use of a larger sample size, a setting of a multicenter, a more focused approach in selecting TCMs and measures that allow the investigation of long term safety of TCMs are recommended for achieving a high-level quality of TCMs evidence for NAFLD to be used to inform clinical practice.

## Data Availability

All data are fully available without restriction.

## Abbreviations

NAFLD: Nonalcoholic fatty liver disease
TCMs: Traditional Chinese medicines
RCTs: Randomized controlled trial
ALB: Albumin
ALP: Alkaline phosphatases
ALT: Aminotransferase
AST: Aspartate aminotransferase
BMI: Body mass index
BU: B-ultrasound
CT: Computed tomography
DAO: Diamine oxidase
FBG: Fasting blood glucose
FINS: Fasting plasma insulin
GGT: Gamma-glutamyl-transpeptidase
GLB: Globulin
GTP: Glutamyl transpeptidase
HDL: High density lipoprotein-cholesterol
HFC: Hepatic fat content
HOMA-IR: Homeostasis model assessment-estimated insulin resistance
hs-CRP: High sensitivity C-reactive protein
IM: Integrative medicine
IRI: Insulin resistance index
L/S ratio: Liver to spleen ratio
LDL: Low density lipoprotein-cholesterol
MRS: Magnetic resonance spectroscopy
TB: Total bilirubin
TC: Total cholesterol
TG: Triglyceride
TNF-α: Tumor necrosis factor-α
VLDL: Very low density lipoprotein
WHR: Waist/hip ratio
WM: Western medicine
